# Dynamic Infrared Thermography Reveals Bilateral Thermal Recovery Asymmetry in Scoliosis

**DOI:** 10.3390/jcm15145720

**Published:** 2026-07-21

**Authors:** Daniel Faizy, Ramazan Beşir Yaman, Seda Nilgün Dumlu, Tuğçe Yavuz, Muhammet Alptekin Kocaoğlu, Akif Albayrak, Hande Argunsah

**Affiliations:** 1 Department of Biomedical Engineering, Faculty of Engineering and Natural Sciences, Acibadem Mehmet Ali Aydinlar University, Istanbul 34752, Turkey; daniel.faizy@live.acibadem.edu.tr (D.F.); ramazan.yaman@live.acibadem.edu.tr (R.B.Y.); seda.dumlu@acibadem.edu.tr (S.N.D.); 2 Graduate School of Natural and Applied Sciences, Biomedical Engineering, Acibadem Mehmet Ali Aydinlar University, Istanbul 34752, Turkey; 3 Department of Orthopaedics and Traumatology, Central Hospital, Istanbul 34752, Turkey; 1tugceyavuz@gmail.com (T.Y.); alptekinkocaoglu@gmail.com (M.A.K.); albayrakakif@gmail.com (A.A.)

**Keywords:** infrared thermography, scoliosis, bilateral asymmetry, bioheat transfer, non-invasive assessment

## Abstract

**Background:** Repeated radiographic monitoring exposes scoliosis patients to cumulative radiation. Dynamic infrared thermography (DIT) may provide a non-invasive alternative by characterizing thermal recovery behavior following controlled cooling. This study investigated whether reheating-based thermographic analysis could reveal scoliosis-related asymmetry. **Methods:** A total of 13 healthy controls and 10 individuals with radiographically confirmed scoliosis were enrolled. Following controlled thoracolumbar cooling, DIT images were acquired. Bilateral paraspinal cold regions were segmented during a 5-min rewarming period. Dynamic thermal asymmetry features were extracted; Sustained Asymmetry Index (SAI) and Dynamic Thermal Asymmetry Index (DTAI) were calculated to quantify asymmetry persistence over time. **Results:** Mean Asymmetry demonstrated significant group-level separation (0.027 ± 0.021 vs. 0.079 ± 0.070, *p* = 0.038, AUC = 0.762). The SAI demonstrated the strongest discriminative performance (0.299 ± 0.114 vs. 0.521 ± 0.143, *p* = 0.0014, AUC = 0.900; sensitivity 0.70, specificity 1.00), indicating persistent bilateral thermal divergence throughout reheating. The DTAI was significantly higher in the scoliosis group (−0.405 ± 0.407 vs. 0.527 ± 0.878, *p* = 0.0084, AUC = 0.831). Inspection of recovery curves revealed distinct thermal recovery phenotypes, including persistent asymmetry, delayed asymmetry peaks, and near-normal thermal recovery patterns. No significant correlation was observed between DTAI and Cobb angle (ρ = 0.183, *p* = 0.611). **Conclusions:** DIT combined with quantitative thermal recovery analysis can reveal bilateral thermo-physiological asymmetry associated with scoliosis. Persistence-based thermal recovery metrics, particularly the SAI, demonstrated strong discriminative performance. These findings support the potential utility of DIT recovery analysis as a non-invasive physiological biomarker framework for scoliosis assessment.

## 1. Introduction

Scoliosis is a three-dimensional spinal deformity characterized by lateral curvature, vertebral rotation, and postural asymmetry [1,2,3,4]. Adolescent Idiopathic Scoliosis (AIS) affects approximately 1–3% of children aged 10–16 years and frequently requires long-term clinical monitoring during periods of rapid growth [1,2,3,4]. Radiographic imaging remains the gold standard for scoliosis diagnosis and Cobb angle assessment, the standard radiographic measure of lateral spinal curvature magnitude, with curves of 10° or greater considered clinically significant; however, repeated exposure to ionizing radiation during follow-up represents a major clinical concern in this predominantly adolescent population [1,5]. To reduce dependence on repeated radiographic evaluation, increasing attention has been directed toward non-invasive and radiation-free assessment methods [2,3,4]. Infrared thermography (IRT) has emerged as a promising complementary tool because skin temperature distribution reflects underlying muscle activity, tissue perfusion, and biomechanical asymmetry and has become increasingly recognized as a non-invasive imaging modality across a broad range of biomedical and musculoskeletal applications [5,6,7,8,9,10]. Previous studies have demonstrated that static thermographic asymmetries may differentiate scoliotic and healthy individuals and may provide information regarding brace effectiveness and postural imbalance. Angrisani et al. reported that thermography can be used for real-time assessment of scoliosis braces [6], while Lubkowska and Gajewska demonstrated temperature asymmetries along the paraspinal musculature in scoliosis patients [7]. Furthermore, Roggio et al. concluded in a recent systematic review that thermography is a valid method to assess scoliosis-related thermal asymmetry, while noting that standardized acquisition protocols remain underdeveloped [11]. This recommendation is consistent with recent literature identifying protocol harmonization as one of the key barriers to wider clinical implementation of medical infrared thermography [8,12,13]. Kwok et al. also demonstrated the feasibility of thermography for scoliosis screening applications [14].

Despite these promising findings, most previous thermographic studies in scoliosis have relied on static thermal images acquired at a single time point. Static imaging cannot fully characterize dynamic physiological mechanisms such as perfusion, vasomotor responses, or tissue heat-transfer behavior. Dynamic infrared thermography (DIT) addresses this limitation by introducing a controlled thermal stimulus followed by continuous monitoring of the reheating process. Rewarming dynamics provide physiologically rich information reflecting dermal perfusion, autonomic vasomotor activity, and metabolic heat generation [15,16,17]. Recent systematic reviews have further highlighted the growing clinical application of dynamic infrared thermography for assessing tissue perfusion, vascular function, and postoperative tissue viability, supporting its potential as a functional physiological imaging modality beyond conventional static thermal assessment [8,13]. Foundational studies by Wilson et al. demonstrated that dynamic thermal recovery curves can be modeled to estimate physiological parameters such as perfusion rate, tissue conductivity, and thermal time constants [18,19]. Dynamic thermography additionally enables extraction of parameters including thermal recovery rate, maximum temperature recovery, and bilateral asymmetry in rewarming behavior. Compared with static imaging, DIT may reduce confounding effects related to transient muscle activation and subcutaneous fat thickness, thereby providing deeper insight into neuromuscular and perfusion-related asymmetry. However, despite these advantages, dynamic thermography has not been systematically investigated for scoliosis assessment, representing a significant gap in the current literature. 

The physiological rationale for dynamic thermographic assessment in scoliosis is supported by bioheat transfer theory. Heat transfer within biological tissues occurs through conduction, blood perfusion–mediated convection, metabolic heat generation, and infrared radiation emission [10]. According to the classical Pennes bioheat equation, tissue temperature evolution is influenced by thermal conductivity, perfusion rate, and metabolic heat production [20]. In scoliosis, vertebral rotation and asymmetric paraspinal muscle activation may alter regional perfusion and tissue composition, producing side-dependent differences in thermal recovery following cooling [21,22]. Regions with higher perfusion and muscle activity are expected to rewarm more rapidly, whereas low-perfusion or mechanically altered tissues may exhibit delayed recovery [23]. The thermal recovery process typically follows an exponential behavior characterized by a thermal time constant (τ), which reflects tissue perfusion and conductivity. Consequently, bilateral differences in rewarming trajectories may serve as indirect markers of scoliosis-related biomechanical and physiological asymmetry also suggested in literature [24]. 

Therefore, this study proposes a reheating-based dynamic thermography framework for detecting bilateral thermal asymmetry associated with scoliosis. Following controlled cooling of the thoracolumbar region, sequential infrared images were analyzed to extract bilateral recovery features reflecting asymmetry in thermal dynamics. We hypothesized that individuals with scoliosis would exhibit greater bilateral asymmetry during thermal recovery compared with healthy controls due to differences in paraspinal muscle activation, tissue composition, and perfusion associated with spinal deformity. 

## 2. Materials and Methods

### 2.1. Participants

A total of 23 participants were enrolled in this comparative study, including 13 healthy controls and 10 individuals with radiographically confirmed scoliosis (Table 1). The healthy control group had a mean age of 21.5 ± 2.0 years, body weight of 68.8 ± 18.0 kg, height of 172.3 ± 10.2 cm, and BMI of 22.4 ± 3.2 kg/m^2^. The scoliosis group had a mean age of 18.9 ± 2.7 years, body weight of 74.8 ± 14.9 kg, height of 175.6 ± 12.0 cm, and BMI of 24.0 ± 3.0 kg/m^2^. Participants in the scoliosis group had Cobb angles ranging from 10° to 41° (mean: 19.9 ± 9.3°). Healthy participants reported no history of spinal deformity, back surgery, or musculoskeletal disorders affecting posture or gait.

Inclusion criteria were: (1) age between 15 and 24 years, (2) ability to maintain an upright standing posture during the recording period, and (3) radiographically confirmed scoliosis diagnosis for the scoliosis group. Exclusion criteria included dermatological conditions affecting the back region, fever or systemic inflammatory conditions, previous spinal surgery, and use of medications known to alter peripheral circulation. Participants with body mass index (BMI) values above 30 kg/m^2^ were excluded to minimize thermal distortion associated with excessive subcutaneous adipose tissue [16]. All participants provided written informed consent prior to participation. The study protocol was approved by the institutional ethics committee of Acıbadem Mehmet Ali Aydınlar University (approval date: 26 March 2026, approval number: 2026-06/234) and conducted in accordance with the Declaration of Helsinki.

### 2.2. Equipment and Study Protocol

Thermal recordings were acquired using a FLIR E8 infrared thermal camera (FLIR Systems Inc., Wilsonville, OR, USA) with a thermal sensitivity of ≤0.06 °C, a spatial resolution of 320 × 240 pixels, and a spectral range of 7.5–13 μm. The camera was mounted on a fixed tripod at approximately 1.2–1.5 m from the participant and aligned perpendicular to the posterior thoracolumbar region. Emissivity was standardized at 0.98 for human skin [7]. All experiments were conducted in a temperature-controlled laboratory environment (22 ± 1 °C; relative humidity: 45–55%) to minimize environmental effects on skin temperature. Participants underwent a 10-min acclimatization period before image acquisition. Thermal image preprocessing and feature extraction were performed in MATLAB (Version: Version R2026a, MathWorks, Natick, MA, USA). Initial thermal frame standardization and grayscale export were conducted using FLIR Studio (Version 7.7.16) software with MSX enhancement disabled to preserve raw thermal information.

Participants stood in a relaxed upright position with the thoracolumbar region fully exposed following a 10-min acclimatization period under controlled laboratory conditions (ambient temperature: 22 ± 1 °C; relative humidity: 45–55%). Prior to data acquisition, the skin surface was cleaned using alcohol-free wipes to minimize emissivity-related artifacts and improve thermal consistency. Controlled thermal perturbation was then induced using a gel-based cooling pad symmetrically applied over the thoracolumbar region for 60 s. To ensure consistent bilateral thermal contact and minimize pressure variability, the cooling pad was secured using a custom stabilization brace. The cooling protocol aimed to reduce the skin surface temperature by approximately 4–6 °C below baseline. Immediately following pad removal, thermal acquisition was initiated and images were recorded at 10-s intervals during a 5-min natural rewarming period, yielding approximately 30 sequential frames per participant. Participants were instructed to remain motionless throughout.

### 2.3. Image Processing

All thermal image sequences were standardized in FLIR Studio using grayscale export with MSX enhancement disabled to preserve raw thermal intensity information. Sequences were then analyzed in MATLAB (MathWorks, Natick, MA, USA). Sequential translational motion correction was applied frame-by-frame using intensity-based translation estimation (imregtform, monomodal optimizer), with registration accuracy evaluated using structural similarity (SSIM), normalized cross-correlation, mean absolute error, and edge-based NCC metrics.

Following motion correction, torso segmentation and anatomical midline estimation were performed to establish a stable bilateral reference axis. The torso boundary was manually initialized on the first post-cooling frame and refined using binary masking and morphological smoothing. The anatomical midline was estimated geometrically from the left and right torso boundaries and smoothed using a Gaussian filter across the thoracolumbar region. Bilateral paraspinal regions of interest (ROIs) were automatically identified by cold-cluster segmentation: pixels below a subject-specific percentile intensity threshold (30–50th percentile of torso-region pixel intensities) applied to the first post-cooling frame. The threshold was selected to ensure complete bilateral capture of the cooled paraspinal regions while minimizing inclusion of surrounding non-cooled tissue. Importantly, once determined, the same threshold was maintained throughout the entire thermal recovery sequence for each participant without further operator adjustment, thereby ensuring consistent ROI tracking across all subsequent frames.

Five dynamic thermal asymmetry features were subsequently calculated for each participant: AI Signed, AI Unsigned, Mean Asymmetry, Max Asymmetry, and Rate Difference. AI Signed represented the directional thermal dominance between the left and right sides, whereas AI Unsigned quantified the magnitude of bilateral divergence independent of laterality. Mean Asymmetry reflected the average difference in bilateral thermal recovery behavior over time, Max Asymmetry captured the maximum transient divergence observed during reheating, and Rate Difference quantified asymmetry in early rewarming slopes between sides. The early-slope Rate Difference feature is conceptually grounded in prior dynamic thermography rewarming analysis [10,12], and the bilateral ratio metrics draw on asymmetry index conventions established in prior scoliosis thermography studies [3,4,5].

Five scalar features were extracted from the bilateral thermal recovery curves. L(*n*) and R(*n*) denote the mean grayscale intensity of the left and right cold-cluster regions at frame *n*, respectively, where *n* = 1, 2, …, *N* (*N* = 30). Recovery increments were defined as ΔL(*n*) = L(*n*) − L(1) and ΔR(*n*) = R(*n*) − R(1).

AI Signed: The signed asymmetry index was defined as:

$$\;\;A(n)=\frac{L(n)-R(n)}{|L(n)|+|R(n)|}$$

$$\;\;AI_{Signed}=\frac{1}{N}\sum_{n=1}^{N}A(n)$$

Positive values indicate left-side thermal dominance, whereas negative values indicate right-side dominance.

AI Unsigned: This feature quantifies the overall magnitude of bilateral thermal asymmetry independent of laterality.

$$AI_{Unsigned}=\frac{1}{N}\sum_{n=1}^{N}|A(n)|$$

Mean Asymmetry:

$$\;Mean\;Asymmetry=\frac{1}{N}\sum_{n=1}^{N}\frac{|\Delta L(n)-\Delta R(n)|}{|\Delta L(n)|+|\Delta R(n)|+\varepsilon }$$

where ε = 1 × 10^−6^ is a small stabilizing constant to prevent zero-division (negligible relative to typical denominator magnitudes). This feature reflects the average bilateral divergence in thermal recovery dynamics over time.

Max Asymmetry: This parameter captures the maximum transient asymmetry observed during the reheating sequence.

$$Max\;Asymmetry=\underset{n}{max}\frac{|\Delta L(n)-\Delta R(n)|}{|\Delta L(n)|+|\Delta R(n)|+\varepsilon }$$

Rate Difference: This feature represents asymmetry in the initial rewarming rate between the left and right paraspinal regions. Early recovery slopes were estimated from the first five frames:

$$S_{L}=slope\left(\Delta L\left(1:5\right)\right),S_{R}=slope\left(\Delta R\left(1:5\right)\right)$$

$$Rate\;Difference=|S_{L}-S_{R}|$$

Sustained Asymmetry Index (SAI): Proposed in the present study as a novel index of asymmetry temporal persistence, SAI quantifies the temporal persistence of bilateral thermal asymmetry during the rewarming sequence. A ratio approaching 1.0 indicates that the asymmetry remains elevated throughout the recording period, producing a plateau-like recovery pattern characteristic of sustained structural perfusion asymmetry. In contrast, lower SAI values indicate transient asymmetry peaks that rapidly resolve over time, consistent with incidental or functional thermal divergence rather than persistent physiological imbalance.

SAI=Mean Asymmetry/Max Asymmetry

To obtain a more robust group-level representation of overall thermo-physiological asymmetry, a composite parameter termed the Dynamic Thermal Asymmetry Index (DTAI) was additionally calculated. The DTAI integrates three complementary features: Mean Asymmetry (sustained bilateral magnitude), Rate Difference (early rewarming kinetics), and SAI (temporal persistence). Because the present study was exploratory, the normalization parameters were derived from the study cohort itself. Consequently, the resulting DTAI should be interpreted as a proof-of-concept composite metric that requires independent external validation before routine clinical application. Prior to DTAI computation, these three features underwent z-score normalization because the numerical ranges and variances of the extracted features differed substantially. For each feature, normalization was performed using the cohort mean and standard deviation:

$$Z=\frac{x-\mu }{\sigma }$$

where *x* represents the original feature value, *μ* the cohort mean, and *σ* the cohort standard deviation. This procedure standardized all parameters onto a common scale with mean 0 and standard deviation 1, preventing high-magnitude variables from disproportionately influencing the composite index. The normalized values of Mean Asymmetry, Rate Difference, and SAI were then averaged using equal weighting to generate a single DTAI score for each participant:

$$DTAI=\frac{Z(Mean\;Asymmetry)+Z(Rate\;Difference)+Z(SAI)}{3}$$

AI Signed was excluded from the composite index because its directional laterality can cancel magnitude under averaging. AI Unsigned was excluded because it was substantially correlated with Rate Difference (verified r = 0.72). The three selected features were mutually low-correlated (r = 0.52, 0.65, and 0.15 for the three pairings), supporting independent contributions to the composite.

### 2.4. Statistical Analysis

Statistical analyses were performed in MATLAB (MathWorks, Natick, MA, USA). Group differences between healthy controls and scoliosis participants were assessed using two-tailed Mann–Whitney U tests with the asymptotic method without continuity correction and observed distributional skew; Shapiro–Wilk testing confirmed non-normal distributions for several features (*p* < 0.05). The nominal significance threshold was α = 0.05. Because the study is exploratory, results are also evaluated against a Bonferroni-corrected threshold for six features.

Descriptive statistics are reported as mean ± standard deviation. Discriminative performance was evaluated using receiver operating characteristic (ROC) analysis, including area under the curve (AUC), sensitivity, and specificity at the Youden-optimal threshold. Effect size was described via group mean difference and AUC (equivalent to rank-biserial correlation); confidence intervals for AUC were not computed given the exploratory sample size. Feature values were extracted automatically in MATLAB and organized into a 23 × 6 feature matrix comprising AI Signed, AI Unsigned, Mean Asymmetry, Max Asymmetry, Rate Difference, and SAI. Ninety-five percent confidence intervals (95% CIs) for the AUC values were estimated using nonparametric bootstrap resampling (10,000 iterations).

The relationship between thermographic asymmetry and scoliosis severity was evaluated using Spearman rank correlation analysis (ρ) between the DTAI and Cobb angle. All reported *p*-values were two-tailed. A post hoc power analysis was additionally performed for SAI, which showed the strongest group separation. Based on the observed group means and standard deviations (healthy controls: 0.299 ± 0.114; scoliosis: 0.521 ± 0.143), the effect size was large (Cohen’s d = 1.745; Hedges’ g = 1.682). Using a two-tailed independent-samples comparison with α = 0.05, the achieved statistical power was estimated as 97.7%. Nevertheless, because of the small sample size and exploratory design, these findings should be interpreted as preliminary and require confirmation in larger prospectively powered cohorts.

## 3. Results

### 3.1. Group-Level Feature Comparisons

**Figure 1:**
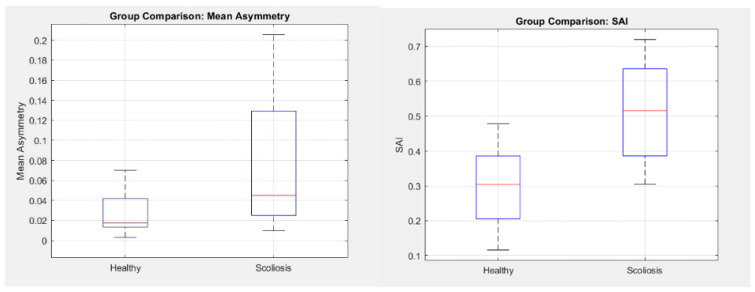
Group-level comparison of the two features with the strongest group separation in healthy controls (*n* = 13) and scoliosis subjects (*n* = 10). (**Left**) Mean Asymmetry differs nominally between groups (*p* = 0.038, AUC = 0.762) but does not survive Bonferroni correction. (**Right**) SAI shows clean separation between groups, with non-overlapping interquartile ranges (*p* = 0.0014, AUC = 0.900) and survives Bonferroni correction (α/6 = 0.0083). Boxes show the interquartile range, central red lines indicate medians, and whiskers extend to the most extreme non-outlier values. No outliers are present in either group for either feature.

Individual and group-level feature values are summarized in Table 2. Among the evaluated parameters, SAI demonstrated the strongest group separation (*p* = 0.0014, AUC = 0.900), with a sensitivity of 0.70 and specificity of 1.00 at the optimal threshold (SAI = 0.493). Scoliosis participants exhibited substantially higher SAI values (0.521 ± 0.143) than healthy controls (0.299 ± 0.114), indicating that bilateral thermal divergence persisted throughout the 5-min rewarming period rather than resolving rapidly. As illustrated in Figure 1, SAI showed clear separation between groups with non-overlapping interquartile ranges and remained significant following Bonferroni correction for six comparisons (α/6 = 0.0083).

Mean Asymmetry also differed between groups (0.079 ± 0.070 vs. 0.027 ± 0.021; *p* = 0.038, AUC = 0.762), with scoliosis participants exhibiting approximately three-fold higher values than healthy controls. However, this feature did not remain significant after Bonferroni correction and should therefore be considered a hypothesis-generating finding. The remaining primary features (AI Unsigned, AI Signed, Max Asymmetry, and Rate Difference) did not reach statistical significance individually, although all demonstrated directionally elevated values in the scoliosis group.

Group-level DTAI values were significantly elevated in the scoliosis cohort (0.527 ± 0.878) compared with healthy controls (−0.405 ± 0.407) (Mann–Whitney U test, *p* = 0.0084). ROC analysis demonstrated good discriminative performance (AUC = 0.831). At the optimal threshold (DTAI = 0.16), sensitivity was 0.60 and specificity was 0.92. The highest DTAI scores were observed in subjects S01 (1.484), S06 (1.810), and S08 (1.447), all of whom demonstrated persistent or delayed bilateral thermal divergence patterns. In contrast, S02 (−0.465) and S05 (−0.130) exhibited DTAI values overlapping with the healthy range. Spearman rank correlation analysis demonstrated no significant association between DTAI and Cobb angle within the scoliosis group (ρ = 0.183, *p* = 0.611). The SAI demonstrated the strongest discriminative performance (AUC = 0.900, 95% CI: 0.746–1.000), with a sensitivity of 0.70 and specificity of 1.00 at the optimal threshold. ROC analysis demonstrated good discriminative performance for DTAI (AUC = 0.831, 95% CI: 0.629–0.975).

### 3.2. Representative Thermal Recovery Patterns

**Figure 2:**
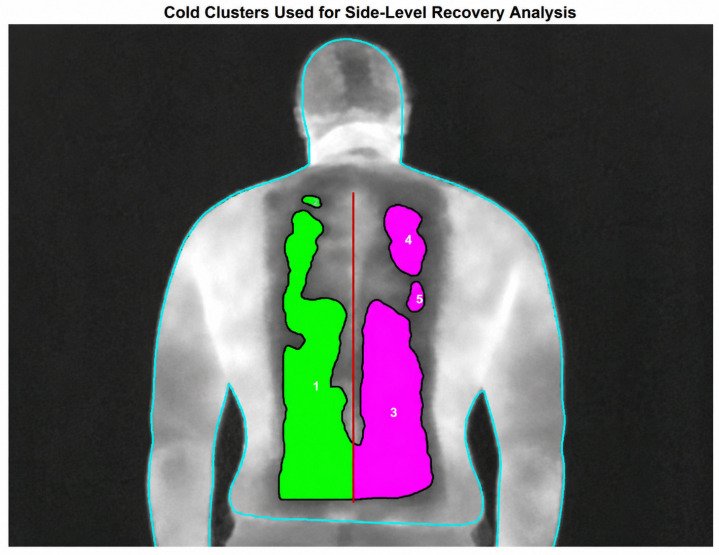
Representative example of automated bilateral cold-cluster segmentation in a healthy participant (H01) following controlled thoracolumbar cooling. Green and magenta regions indicate left- and right-side cold clusters, respectively, with the estimated anatomical midline (red line) used for bilateral ROI assignment, Cyan: Body contour outline.

**Figure 3:**
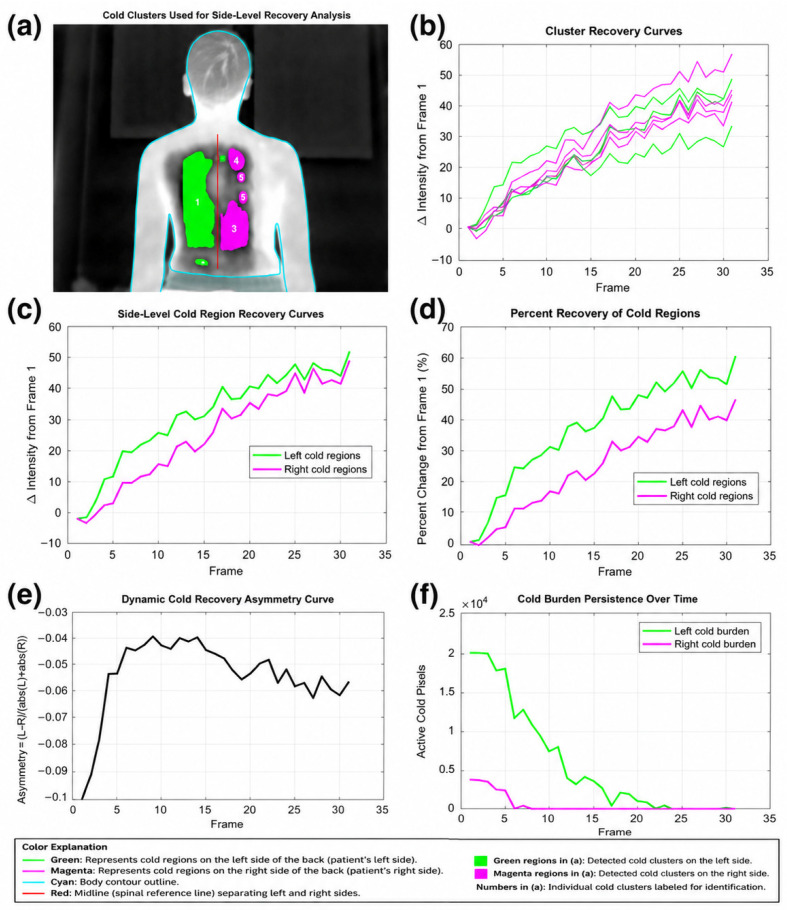
Dynamic thermal recovery analysis workflow for a representative scoliosis subject (S01). (**a**) Automated cold-cluster segmentation on the first post-cooling frame. (**b**) Individual cluster-level recovery curves (frame-wise ΔIntensity). (**c**) Aggregated side-level recovery curves for left (green) and right (magenta) paraspinal regions. (**d**) Normalized percent recovery curves. (**e**) Dynamic asymmetry curve across all frames, peaking at approximately 0.30 at frame 6. (**f**) Cold burden persistence (active cold pixels) for left and right sides across all frames.

Visual inspection of the thermal recovery curves revealed multiple distinct recovery phenotypes within the scoliosis cohort, including persistent asymmetry, delayed asymmetry peaks, and near-normal thermal recovery patterns. Representative examples of the thermal recovery workflow and side-level recovery behavior are shown in Figure 2 and Figure 3a. Representative thermographic images and recovery curves are presented in the main manuscript, while the complete thermographic image series and corresponding thermal recovery graphs for all study participants are provided in the Supplementary Materials.

Subjects S01 and S06 demonstrated the strongest persistent bilateral asymmetry patterns. S01 exhibited elevated values across multiple features (AI Unsigned = 0.399, Mean Asymmetry = 0.115, Rate Difference = 4.10) despite a moderate Cobb angle of 14°, with incomplete convergence of the asymmetry curve throughout the 300-s recording period. Supplementary File indicates that S01 was a young female participant with a relatively low body mass index, suggesting that the observed thermal asymmetry was unlikely to be attributable to body composition alone. S06, the most severe scoliosis case (Cobb angle = 41°), demonstrated the highest Mean Asymmetry value in the dataset (0.206) together with a persistently negative, diverging asymmetry curve throughout the 300-s window, a trajectory qualitatively distinct from S01, potentially reflecting entrenched perfusion asymmetry at high curve magnitudes. Individual clinical characteristics provided in Supplementary File further show that S06 presented with one of the largest spinal deformities in the cohort, supporting the possibility that prolonged thermal asymmetry may be associated with greater structural involvement.

A second phenotype was observed in S08, which demonstrated the highest AI Unsigned value in the cohort (0.616). Unlike S01, the asymmetry peak occurred later in the reheating sequence (frames 13–15), indicating delayed thermal divergence. As shown in Supplementary File, S08 had the highest body weight and among the highest BMI values in the cohort (105 kg; BMI 27.9 kg/m^2^), suggesting that individual anthropometric characteristics may have influenced local heat transfer and rewarming kinetics.

In contrast, subjects S02 and S05 exhibited thermographic profiles overlapping entirely with the healthy range despite confirmed scoliosis diagnoses, representing two mechanistically distinct compensation pathways. S02 demonstrated near-symmetric cold-cluster distributions and parallel bilateral recovery curves throughout (structural compensation). S05 showed an initially asymmetric cold distribution but crossing recovery curves at approximately frame 7, with the right side ultimately overtaking the left (dynamic functional compensation), so that the net time-averaged asymmetry remained low. The individual profiles in Supplementary File suggest that thermographic recovery patterns may not always mirror structural deformity severity, supporting the presence of compensatory physiological mechanisms in some scoliosis cases.

Healthy participants generally demonstrated symmetric thermal recovery behavior characterized by near-parallel bilateral recovery curves, rapid bilateral convergence, and minimal persistent asymmetry throughout the reheating period.

## 4. Discussion

This study demonstrates that controlled cooling followed by quantitative rewarming analysis can detect bilateral thermal asymmetry associated with scoliosis. From a biological perspective, the persistent paraspinal thermal divergence observed in the scoliosis cohort reflects differences in regional perfusion, metabolic heat production, and tissue thermal conductivity between the concave and convex sides of the deformed spine; asymmetries that are structurally sustained by the altered muscle fiber composition, vascular distribution, and neuromuscular activation patterns characteristic of AIS [20,21,22]. The Pennes bioheat framework provides a plausible mechanistic basis for this finding: vertebral rotation and asymmetric paraspinal muscle activation alter regional perfusion (ωb) and metabolic heat generation (Qm), producing side-dependent rewarming trajectories [20]. Histological evidence indicates that paraspinal muscle fibers are larger on the convex side of the scoliotic apex [21], and EMG analyses demonstrate higher muscle activation on the convex relative to the concave side [22]. These are asymmetries in tissue composition and perfusion that would produce precisely the bilateral thermal recovery differences quantified here. IVIM-MRI perfusion imaging has independently demonstrated asymmetric paraspinal muscle blood flow after exercise in AIS patients [23], providing direct imaging corroboration of the perfusion-driven mechanism proposed in the present study. These findings are consistent with recent reviews emphasizing that infrared thermography primarily reflects physiological tissue function rather than structural morphology, supporting its use as a complementary imaging modality in musculoskeletal assessment [8,9,10,13,24]. 

The strongest-performing individual parameter was the SAI, which quantified the persistence of bilateral thermal divergence throughout the reheating sequence. Unlike transient asymmetry peaks, elevated SAI values indicated that the bilateral difference remained consistently elevated over time rather than rapidly resolving. Critically, SAI (*p* = 0.0014) survived Bonferroni correction (α/6 = 0.0083), whereas DTAI (*p* = 0.0084) approached but did not strictly meet the corrected threshold, suggesting that SAI represents the most statistically robust signal in the present dataset. Mean Asymmetry (*p* = 0.038) does not survive this correction entirely and should be treated as a hypothesis-generating finding pending independent replication. The specificity of SAI (1.00) is particularly notable: no healthy subject exceeded SAI = 0.493, suggesting that sustained bilateral thermal divergence is a strong negative predictor of a healthy spine in this cohort.

The lack of a significant correlation between DTAI and Cobb angle indicates that the proposed thermographic biomarkers are not simply surrogate measures of structural curve severity. Instead, they appear to reflect physiological characteristics of the paraspinal tissues that are only partially related to spinal morphology. Dynamic infrared thermography captures processes such as regional blood perfusion, muscle activation, and thermoregulatory responses, which are influenced by multiple biological factors and therefore are not expected to vary linearly with radiographic curve magnitude [22,23,24,25,26]. This observation supports the concept that thermographic assessment provides physiological information distinct from conventional radiographic measurements, reinforcing its value as a complementary functional assessment rather than a direct indicator of structural deformity [8,10,13].

To our knowledge, DTAI is a novel composite biomarker that integrates asymmetry persistence, early rewarming kinetics, and overall recovery divergence into a single z-normalized index. However, given the exploratory design and limited sample size, DTAI should be considered a preliminary proof-of-concept metric. Although the initial findings are encouraging, its diagnostic performance and clinical utility require confirmation in larger, independent, prospectively powered, and preferably multicenter cohorts before it can be considered for routine clinical application.

An additional area of interest is the potential application of dynamic thermography for monitoring treatment response. Future prospective longitudinal studies should evaluate these thermographic biomarkers before and after conservative interventions, such as physiotherapeutic scoliosis-specific exercises or brace treatment. Recent reviews on AIS management have similarly highlighted the need for objective, non-invasive biomarkers capable of monitoring physiological responses to conservative treatment over time [2,3,4]. Correlating changes in thermal recovery metrics with radiographic and clinical outcomes would help determine their potential as radiation-free biomarkers for monitoring treatment response and disease progression.

From a clinical perspective, the proposed thermographic parameters should be viewed as complementary physiological biomarkers rather than surrogate measures of structural deformity. The absence of a significant association between DTAI and Cobb angle suggests that dynamic thermal recovery reflects physiological processes, including regional perfusion, muscle activation, and thermoregulatory behavior, which are not necessarily proportional to radiographic curve magnitude. Consequently, these biomarkers are not intended to replace conventional radiographic assessment but may provide additional functional information that complements structural evaluation. Their greatest potential may lie in radiation-free physiological screening, longitudinal monitoring of tissue-level adaptations, and assessment of treatment-related changes following interventions such as physiotherapeutic scoliosis-specific exercises, brace treatment, or multidisciplinary rehabilitation. Future longitudinal studies should investigate whether changes in these thermographic biomarkers precede or accompany clinical improvement and whether they can serve as sensitive indicators of treatment response or disease progression.

Several limitations should be acknowledged. First, the sample size was relatively small, particularly within the scoliosis cohort, reflecting the exploratory proof-of-concept nature of the present study. Although a post hoc power analysis for the primary outcome (SAI) demonstrated a large observed effect size (Cohen’s d = 1.745; Hedges’ g = 1.682) and high statistical power (97.7%), these estimates should be interpreted cautiously because they are based on the observed data and do not overcome the inherent limitations of a small sample. Consequently, the findings should be considered preliminary, and the generalizability of the proposed thermal recovery biomarkers remains limited. Independent validation in larger, prospectively powered, and preferably multicenter cohorts encompassing a broader spectrum of scoliosis severity is required before clinical implementation can be considered. Second, the scoliosis cohort exhibited substantial heterogeneity in age, body composition, and curve characteristics, which may have contributed to the observed inter-individual variability in thermal recovery patterns. Third, uncontrolled physiological factors, including recent physical activity, muscle conditioning, and autonomic variability, may have influenced thermal recovery behavior and should be prospectively assessed using validated instruments in future studies. Fourth, the study evaluated only short-term thermographic responses using a single cooling protocol and did not include longitudinal follow-up or repeated-session reliability assessments. Although participants with a BMI greater than 30 kg/m^2^ were excluded to reduce the influence of excessive subcutaneous adipose tissue on thermal measurements, residual variability in body composition may still have affected thermal recovery patterns. Future studies should incorporate BMI or, preferably, direct measures of body composition or subcutaneous fat thickness as covariates in multivariable statistical models to determine whether the proposed thermographic biomarkers remain independent of anthropometric characteristics. An additional limitation is that the initial percentile threshold used for cold-cluster segmentation was determined on a subject-specific basis. Although the selected threshold remained fixed throughout the recovery sequence for each participant, the initial selection involved operator judgment and may therefore introduce some degree of subjectivity. Future studies should investigate automated or data-driven segmentation strategies to improve reproducibility, reduce operator dependence, and facilitate clinical implementation. An additional limitation relates to the proposed Dynamic Thermal Asymmetry Index (DTAI). Because the composite index was constructed using z-score normalization based on the entire study cohort, the reported discriminative performance may be optimistically estimated, particularly given the limited sample size. Accordingly, DTAI should be considered a preliminary proof-of-concept biomarker rather than a finalized diagnostic index. Future studies should derive normalization parameters using independent training datasets and subsequently evaluate DTAI in external validation cohorts or through prospective multicenter studies. Such validation will be essential to establish the robustness, generalizability, and clinical utility of the proposed composite biomarker. Finally, the extracted thermal features were derived from grayscale intensity changes rather than absolute calibrated temperature values, which may limit direct physiological interpretation and comparison with established thermographic methodologies.

Despite these limitations, the proposed approach follows an active thermography paradigm that has been successfully applied in vascular assessment, breast oncology, and diabetic foot evaluation, where temporal recovery dynamics following a controlled thermal stimulus reveal physiological differences that may not be detectable using static thermographic measurements. Future studies should focus on substantially expanding the cohort to enable multivariate modeling and severity-stratified analyses, prospectively documenting potential confounding factors such as physical activity levels, and incorporating calibrated radiometric measurements to permit absolute temperature comparisons and bioheat-model-based analyses. Additional work should investigate temporal recovery characteristics beyond magnitude-based asymmetry measures, including trajectory-derived features extracted from asymmetry time series. Longitudinal studies involving patients undergoing physiotherapy may further determine whether dynamic thermographic asymmetry measures can serve as treatment-response biomarkers. Furthermore, repeated-session reliability testing, integration with radiographic and biomechanical assessments, and machine learning–based analysis of temporal recovery patterns may help establish the clinical utility and diagnostic potential of dynamic thermographic recovery biomarkers for non-invasive scoliosis assessment.

The study demonstrates that dynamic thermal recovery analysis can identify measurable bilateral asymmetry patterns associated with scoliosis. The combination of controlled cooling, temporal recovery analysis, and composite asymmetry metrics represents a promising proof-of-concept framework for non-invasive physiological assessment in scoliosis. However, the proposed composite biomarker (DTAI) should be considered preliminary until its reproducibility, diagnostic performance, and clinical utility have been confirmed in larger independent cohorts. Future studies should include larger cohorts, repeated-session reliability analysis, severity-stratified subgroup comparisons, and integration with radiographic and biomechanical measurements. Prospective longitudinal studies should further evaluate these thermographic biomarkers before and after conservative interventions, such as physiotherapeutic scoliosis-specific exercises or brace treatment. Moreover, to facilitate greater methodological consistency across future studies, standardized acquisition protocols should be adopted for dynamic infrared thermography in scoliosis assessment. Recent reviews have continued to identify protocol harmonization as a major prerequisite for broader clinical adoption of infrared thermography [8,9,11,27]. Based on the experience gained from the present study, key considerations include a controlled imaging environment with stable ambient temperature and humidity, an adequate acclimatization period before imaging, standardized participant positioning, a reproducible cooling protocol, immediate initiation of image acquisition following thermal stimulation, consistent camera setup and emissivity settings, frame-by-frame motion correction, and fixed region-of-interest segmentation parameters throughout the recovery sequence. Establishing such standardized procedures will improve reproducibility, facilitate comparison across studies, and support the future clinical translation of dynamic thermographic assessment. Correlating changes in thermal recovery metrics with radiographic and clinical outcomes would help establish their potential as radiation-free biomarkers for monitoring treatment response and disease progression. Future studies should also transition from grayscale intensity–based analysis to calibrated radiometric temperature measurements. Recent clinical thermography reviews have likewise recommended calibrated radiometric imaging and standardized acquisition procedures to improve reproducibility and facilitate clinical translation [8,9,13]. Such an approach would enable direct quantification of tissue temperature, facilitate bioheat-model–based physiological interpretation, improve inter-study comparability, and align dynamic thermographic assessment with established biomedical engineering and clinical thermography standards. Machine learning–based temporal pattern analysis and automated classification approaches may further improve the diagnostic potential of dynamic thermographic recovery biomarkers.

## Figures and Tables

**Table 1:** Demographic and anthropometric characteristics of healthy controls and scoliosis participants.

ID	Age	Gender	Weight (kg)	Height (cm)	BMI	Scoliosis	Cobb Angle (°)
Healthy Controls							
H01	23	Male	120	200	30.0	No	—
H02	22	Male	65	175	21.2	No	—
H03	24	Male	76	175	24.8	No	—
H04	21	Female	50	167	17.9	No	—
H05	20	Female	60	163	22.5	No	—
H06	18	Female	53	158	21.2	No	—
H07	19	Male	71	185	20.7	No	—
H08	22	Male	68	175	22.2	No	—
H09	24	Male	68	173	22.4	No	—
H10	19	Female	55	168	19.5	No	—
H11	23	Female	57	170	19.7	No	—
H12	21	Male	78	170	27	No	—
H13	22	Female	63	171	21.5	No	—
Participants with Scoliosis							
S01	19	Female	75	175	24.0	Yes	14
S02	21	Male	94	182	28.4	Yes	20
S03	15	Male	60	166	21.8	Yes	26
S04	22	Female	63	168	22.3	Yes	23
S05	16	Male	70	180	21.6	Yes	18
S06	15	Male	74	163	27.9	Yes	41
S07	21	Female	65	165	23.9	Yes	23
S08	21	Male	105	194	27.9	Yes	14
S09	20	Female	56	168	19.8	Yes	10
S10	19	Male	86	195	22.6	Yes	10

BMI = body mass index. BMI was calculated as weight (kg) divided by height squared (m^2^).

**Table 2:** Individual subject feature values and group-level statistical comparisons (healthy vs. scoliosis).

Group	ID	Cobb (°)	AI Unsigned	AI Signed	Mean Asym.	Max Asym.	Rate Diff.	SAI	DTAI
Healthy	H01	—	0.0666	−0.0666	0.0226	0.0518	0.324	0.436	−0.287
	H02	—	0.0877	0.0877	0.0185	0.0633	0.318	0.292	−0.601
	H03	—	0.0043	0.0043	0.0116	0.0328	0.061	0.354	−0.610
	H04	—	0.0750	−0.0750	0.0384	0.1583	1.876	0.243	−0.040
	H05	—	0.0377	0.0377	0.0700	0.2304	0.652	0.304	−0.145
	H06	—	0.1709	−0.1709	0.0535	0.1231	2.001	0.435	0.477
	H07	—	0.0515	0.0515	0.0031	0.0084	0.273	0.369	−0.558
	H08	—	0.1028	−0.1028	0.0090	0.0292	0.976	0.308	−0.401
	H09	—	0.0282	−0.0282	0.0176	0.1226	0.774	0.144	−0.744
	H10	—	0.0636	0.0636	0.0171	0.1474	0.293	0.116	−0.967
	H11	—	0.0933	−0.0933	0.0138	0.0661	0.831	0.209	−0.619
	H12	—	0.0262	0.0262	0.0169	0.0868	0.202	0.195	−0.844
	H13	—	0.0850	−0.0850	0.0527	0.1102	0.574	0.478	0.068
Scoliosis	S01	14	0.3992	0.3992	0.1152	0.2986	4.102	0.386	1.484
	S02	20	0.0756	0.0756	0.0171	0.0476	0.350	0.359	−0.465
	S03	26	0.4135	0.4135	0.0540	0.1010	2.257	0.535	0.767
	S04	23	0.3087	−0.3087	0.1293	0.1821	0.432	0.710	0.950
	S05	18	0.0335	0.0335	0.0317	0.0640	0.277	0.495	−0.130
	S06	41	0.3094	−0.3094	0.2056	0.2855	1.504	0.720	1.810
	S07	23	0.0405	−0.0405	0.0097	0.0318	0.383	0.305	−0.607
	S08	14	0.6161	0.6161	0.1668	0.2926	2.011	0.570	1.447
	S09	10	0.0223	0.0223	0.0250	0.0393	0.430	0.636	0.161
	S10	10	0.0189	0.0189	0.0359	0.0728	0.165	0.493	−0.147
Healthy mean ± SD	—	—	0.069 ± 0.043	−0.027 ± 0.078	0.027 ± 0.021	0.095 ± 0.062	0.704 ± 0.611	0.299 ± 0.114	−0.405 ± 0.407
Scoliosis mean ± SD	—	19.9 ± 9.3	0.224 ± 0.213	0.092 ± 0.303	0.079 ± 0.070	0.142 ± 0.112	1.191 ± 1.282	0.521 ± 0.143	0.527 ± 0.878
*p*-value	—	—	0.306	0.306	0.038 *	0.476	0.438	0.0014 **	0.0084 **
AUC	—	—	0.631	0.631	0.762	0.592	0.600	0.900	0.831

* *p* < 0.05; ** *p* < 0.01, two-tailed Mann–Whitney U (uncorrected). SAI = Sustained Asymmetry Index; DTAI = Dynamic Thermal Asymmetry Index; AUC = area under the ROC curve. Cobb angle is not applicable (—) for healthy controls.

## Data Availability

The original contributions presented in this study are included in the article/Supplementary Material. Further inquiries can be directed to the corresponding author.

## Supplementary Materials

The following supporting information can be downloaded at: https://www.mdpi.com/article/10.3390/jcm15145720/s1.

## Abbreviations

The following abbreviations are used in this manuscript:

AIS	Adolescent idiopathic scoliosis
AUC	Area under the ROC curve
BMI	Body mass index
DIT	Dynamic infrared thermography
DTAI	Dynamic Thermal Asymmetry Index
EMG	Electromyography
IPAQ	International Physical Activity Questionnaire
IRT	Infrared thermography
IVIM-MRI	Intravoxel incoherent motion magnetic resonance imaging
MSX	Multi-Spectral Dynamic Imaging
NCC	Normalized cross-correlation
ROC	Receiver operating characteristic
ROI	Region of interest
SAI	Sustained Asymmetry Index
SSIM	Structural similarity index measure

## References

[1] Weinstein S.L., Dolan L.A., Cheng J.C.Y., Danielsson A., Morcuende J.A. (2008). Adolescent idiopathic scoliosis. Lancet.

[2] Jinnah A.H., Lynch K.A., Wood T.R., Hughes M.S. (2025). Adolescent Idiopathic Scoliosis: Advances in Diagnosis and Management. Curr. Rev. Musculoskelet. Med..

[3] Kim H., Chang B.S., Chang S.Y. (2024). Current issues in the treatment of adolescent idiopathic scoliosis: A comprehensive narrative review. Asian Spine J..

[4] Su Y.C., Feng C.K., Yang T.F. (2025). Assessment and Management of Adolescent Idiopathic Scoliosis: From the Perspective of a Physiatrist. Ann. Rehabil. Med..

[5] Hoffman D.A., Lonstein J.E., Morin M.M., Visscher W., Harris B.S., Boice J.D. (1989). Breast cancer in women with scoliosis exposed to multiple diagnostic X rays. J. Natl. Cancer Inst..

[6] Angrisani L., De Benedetto E., Duraccio L., Lo Regio F., Ruggiero R., Tedesco A. (2023). Infrared thermography for real-time assessment of the effectiveness of scoliosis braces. Sensors.

[7] Lubkowska A., Gajewska E. (2020). Temperature distribution of selected body surfaces in scoliosis based on static infrared thermography. Int. J. Environ. Res. Public Health.

[8] Liu Q., Li M., Wang W., Jin S., Piao H., Jiang Y., Li N., Yao H. (2025). Infrared thermography in clinical practice: A literature review. Eur. J. Med. Res..

[9] Kumar B., Jain V., Yadav D.K., Dhua A.K., Goel P., Jain D., Singh S. (2026). Heat for Healing: A Review of Infrared Thermography in Medical Diagnostics and Therapy. J. Med. Phys..

[10] Watts A.T., Boyle A.M., Kutuzov V., Warner C., Staniland T., Barlow G., Sharma H. (2024). Current Use of Infrared Thermography in Orthopaedic and Bone or Joint Trauma Patients-Can We Identify Postoperative Infection? A Narrative Systematic Review. Strateg. Trauma. Limb Reconstr..

[11] Roggio F., Petrigna L., Filetti V., Vitale E., Rapisarda V., Musumeci G. (2023). Infrared thermography for the evaluation of adolescent and juvenile idiopathic scoliosis: A systematic review. J. Therm. Biol..

[12] Kumar P., Gaurav A., Rajnish R.K., Sharma S., Kumar V., Aggarwal S., Patel S. (2021). Applications of thermal imaging with infrared thermography in Orthopaedics. J. Clin. Orthop. Trauma.

[13] Clarys W., Hotome V., Hummelink S., Verspeek S., Verhoeven V., Tjalma W.A.A., Steenackers G., Thiessen F. (2025). Dynamic infrared thermography in free flap surgery: A systematic review. JPRAS Open.

[14] Kwok G., Yip J., Yick K.L., Cheung M.C., Tse C.Y., Ng S.P., Luximon A. (2017). Postural screening for adolescent idiopathic scoliosis with infrared thermography. Sci. Rep..

[15] Ring E.F.J., Ammer K. (2012). Infrared thermal imaging in medicine. Physiol. Meas..

[16] Lee S.L., Lu Y.H. (2014). Modeling of bioheat equation for skin and a preliminary study on a noninvasive diagnostic method for skin burn wounds. Burns.

[17] Jiang L.J., Ng E.Y., Yeo A.C., Wu S., Pan F., Yau W.Y., Chen J.H., Yang Y. (2005). A perspective on medical infrared imaging. J. Med. Eng. Technol..

[18] Wilson S.B., Spence V.A. (1988). A tissue heat transfer model for relating dynamic skin temperature changes to physiological parameters. Phys. Med. Biol..

[19] Wilson S.B., Spence V.A. (1989). Dynamic thermographic imaging method for quantifying dermal perfusion: Potential and limitations. Med. Biol. Eng. Comput..

[20] Pennes H.H. (1948). Analysis of tissue and arterial blood temperatures in the resting human forearm. J. Appl. Physiol..

[21] Shahidi B., Yoo A., Farnsworth C., Newton P.O., Ward S.R. (2021). Paraspinal muscle morphology and composition in adolescent idiopathic scoliosis: A histological analysis. JOR Spine.

[22] Cheung J., Halbertsma J.P., Veldhuizen A.G., Sluiter W.J., Maurits N.M., Cool J.C., van Horn J.R. (2005). A preliminary study on electromyographic analysis of the paraspinal musculature in idiopathic scoliosis. Eur. Spine J..

[23] Federau C., Kroismayr D., Dyer L., Farshad M., Pfirrmann C. (2020). Demonstration of asymmetric muscle perfusion of the back after exercise in patients with adolescent idiopathic scoliosis using intravoxel incoherent motion MRI. NMR Biomed..

[24] Lahiri B.B., Bagavathiappan S., Jayakumar T., Philip J. (2012). Medical applications of infrared thermography: A review. Infrared Phys. Technol..

[25] Fernández-Cuevas I., Bouzas Marins J.C., Arnáiz Lastras J., Gómez Carmona P.M., Piñonosa Cano S., García-Concepción M.Á., Sillero-Quintana M. (2015). Classification of factors influencing the use of infrared thermography in humans: A review. Infrared Phys. Technol..

[26] Schmid A.B., Dyer L., Böni T., Held U., Brunner F. (2010). Paraspinal muscle activity during symmetrical and asymmetrical weight training in idiopathic scoliosis. J. Sport. Rehabil..

[27] Gershenson M., Gershenson J. (2023). Dynamic vascular imaging using active breast thermography. Sensors.

