# Supplementary figures and images for: Dynamic Infrared Thermography Reveals Bilateral Thermal Recovery Asymmetry in Scoliosis
Source: J Clin Med. 2026 Jul 21;15(14):5720. doi: 10.3390/jcm15145720 (PMC13413119; doi:10.3390/jcm15145720)

# SUPPLEMENTARY FILE

## INDIVIDUAL SUBJECT FIGURES

**H01**

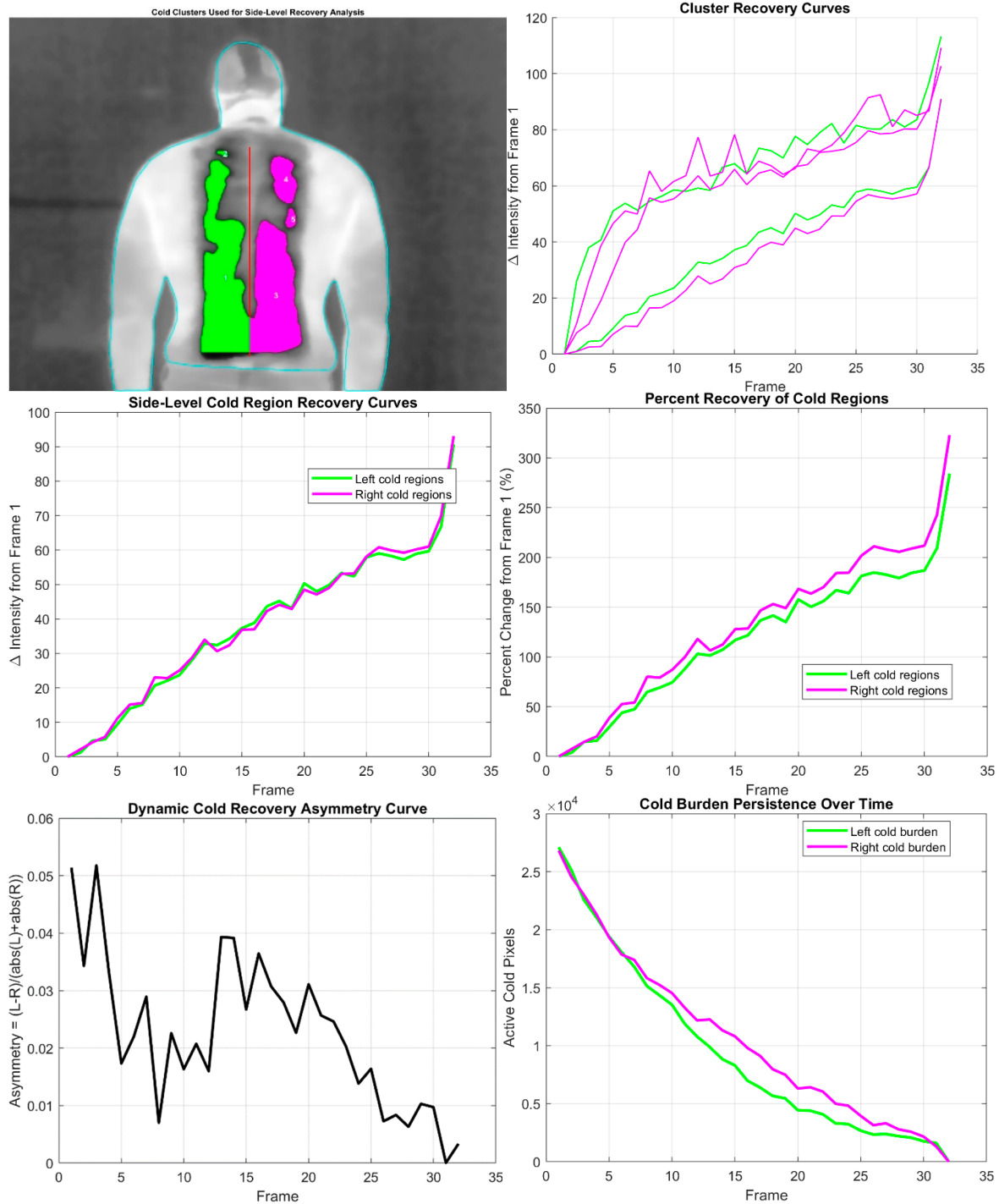

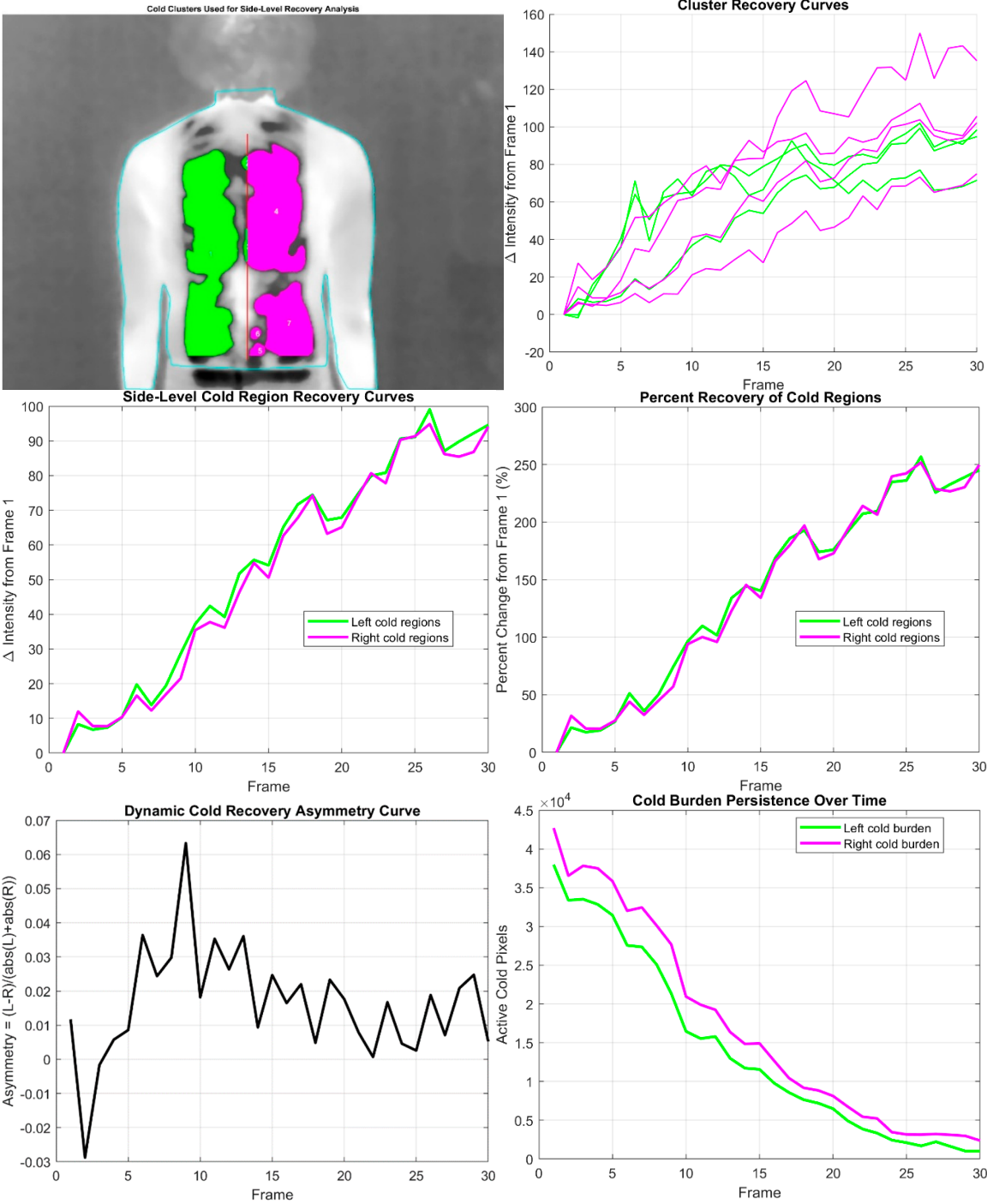

# H03

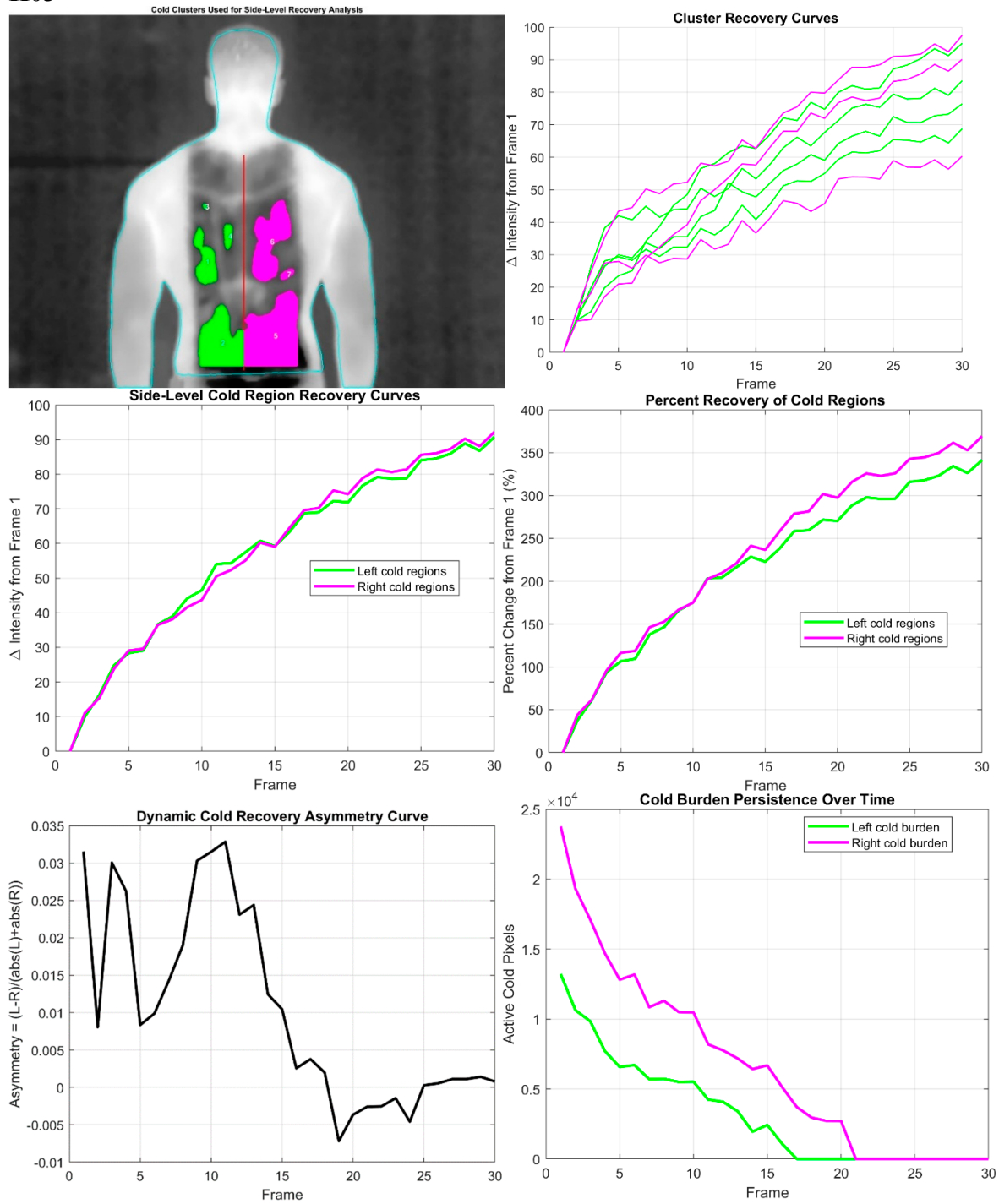

## H04

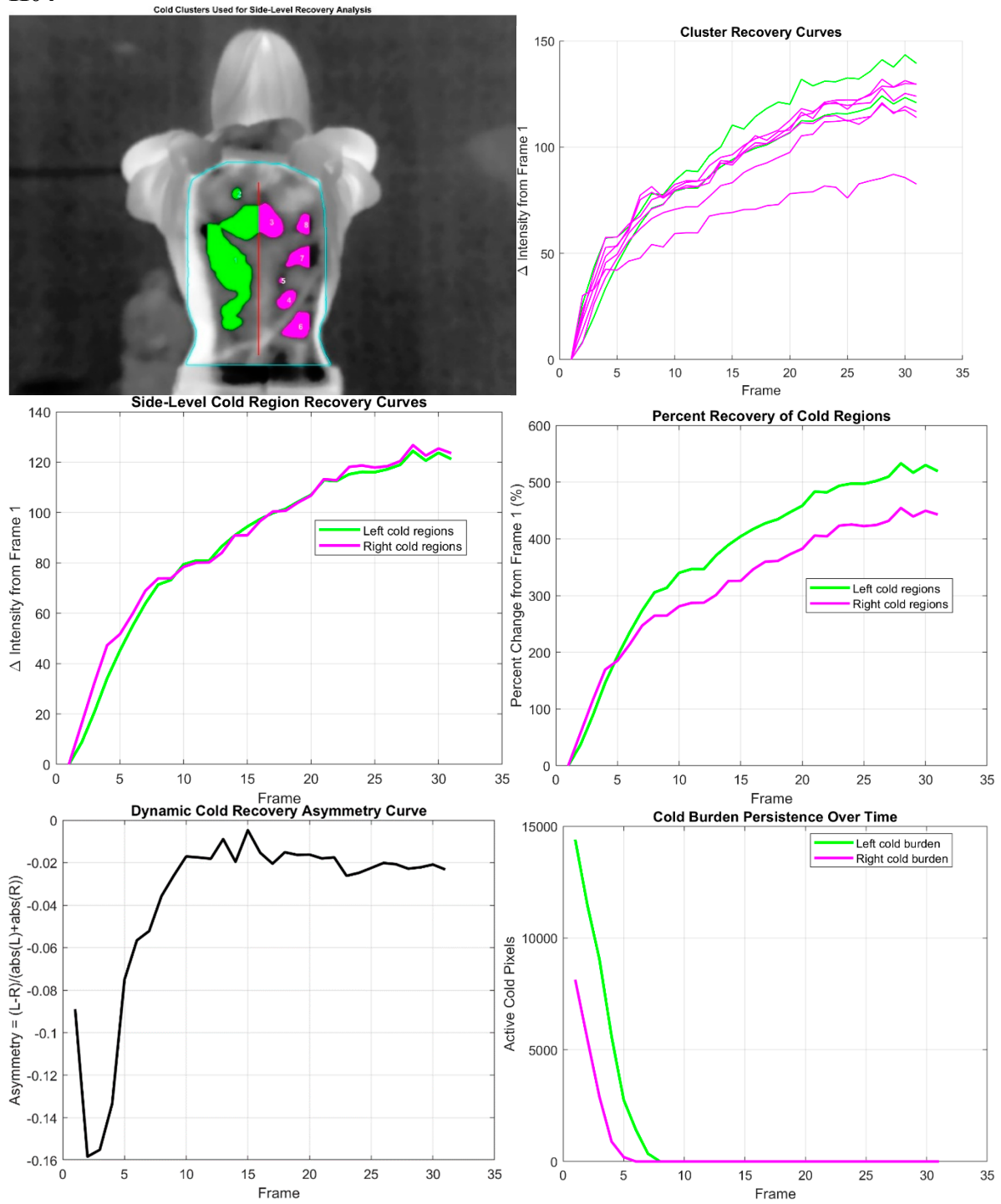

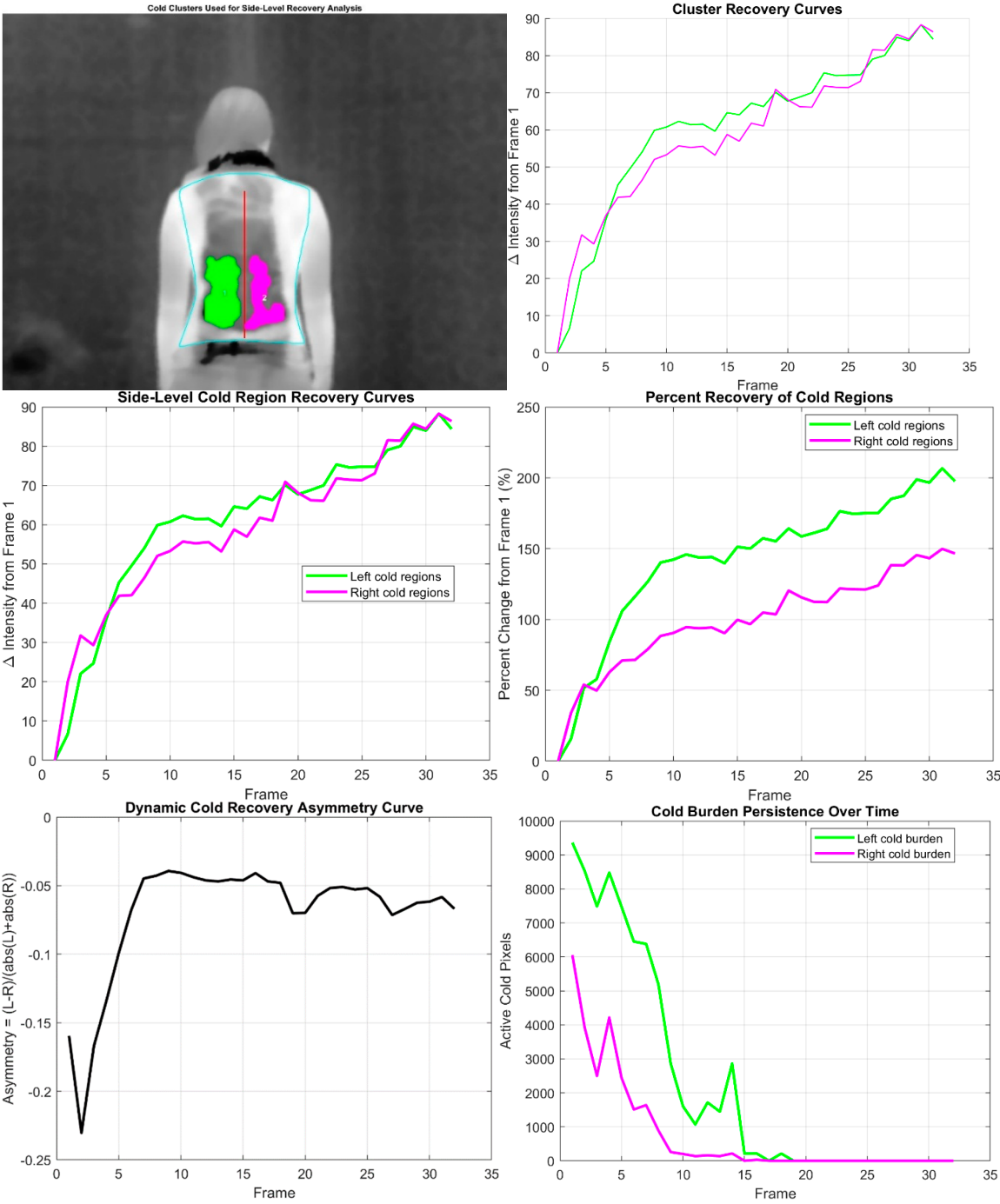

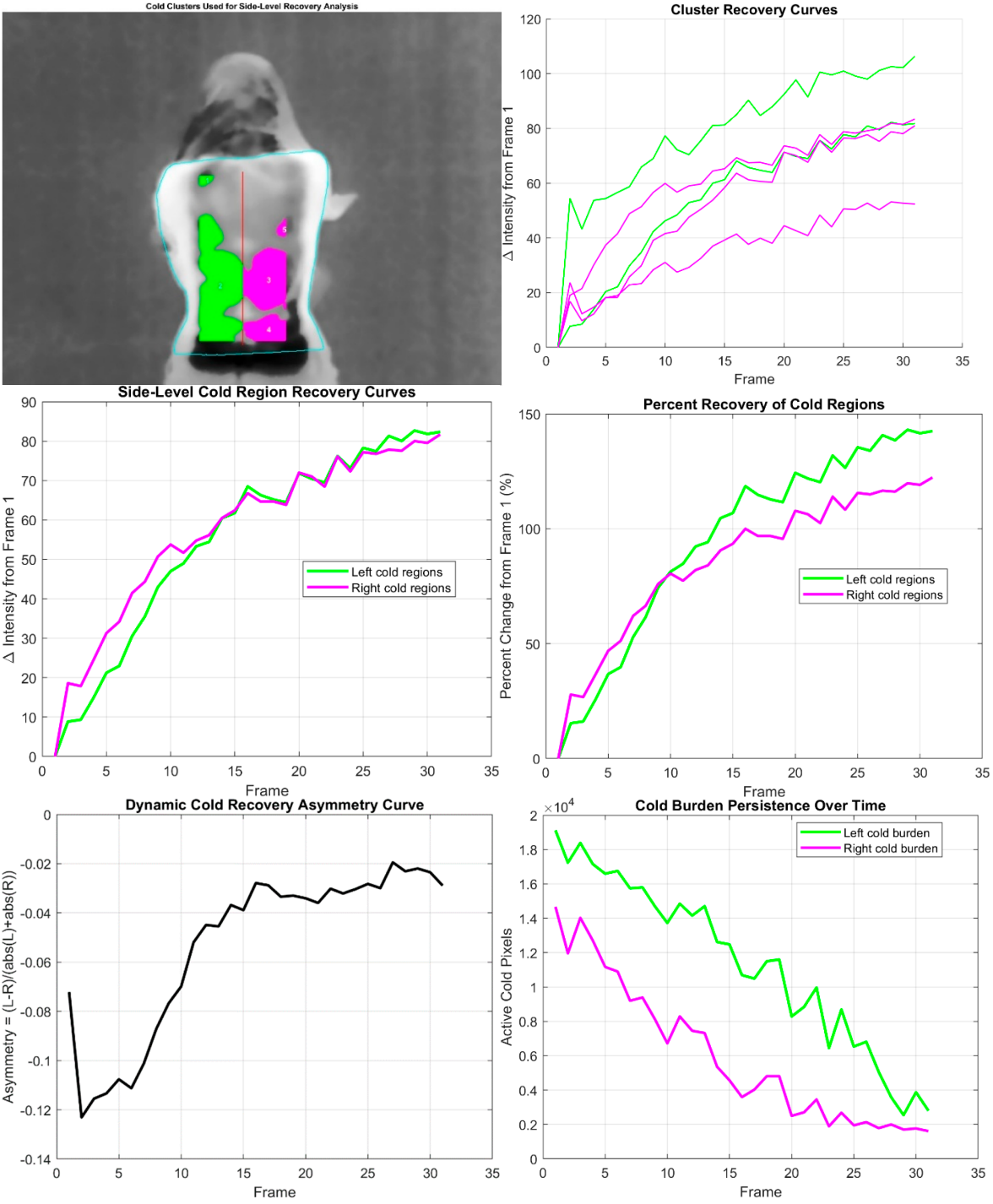

H07

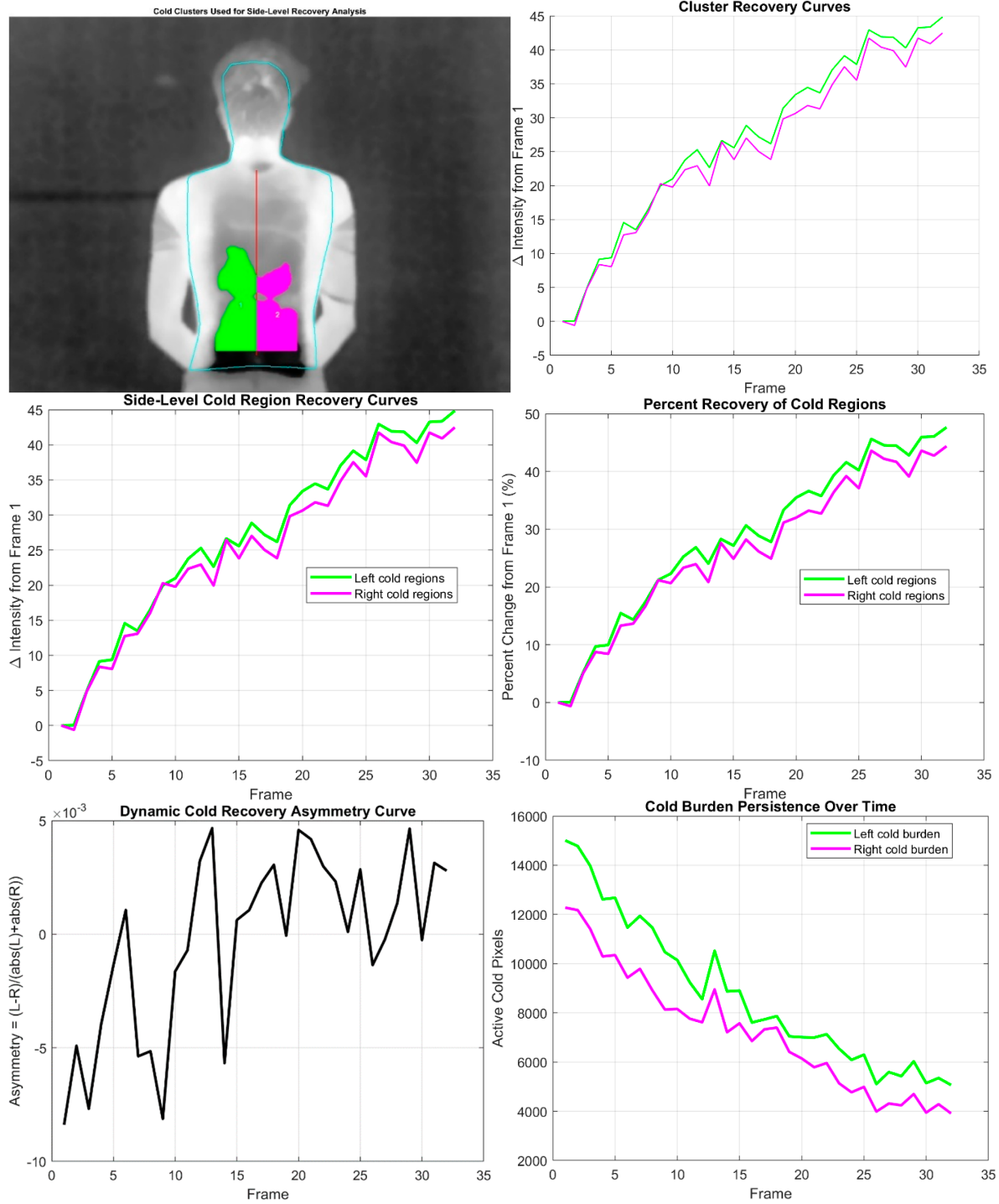

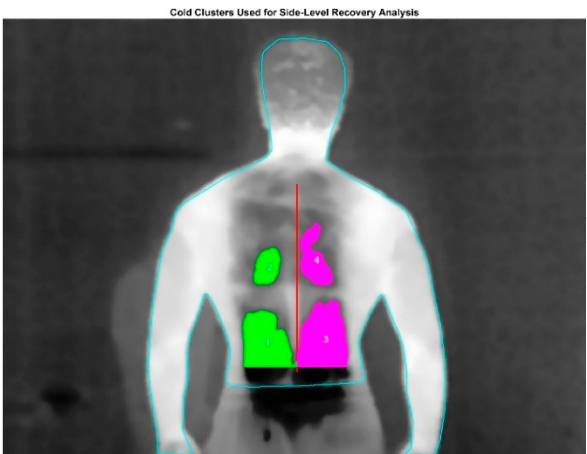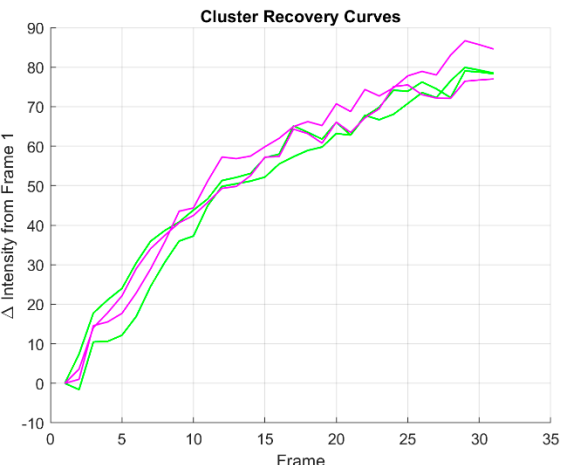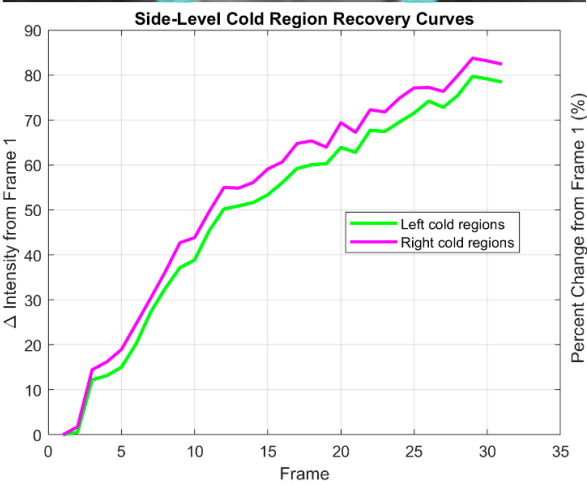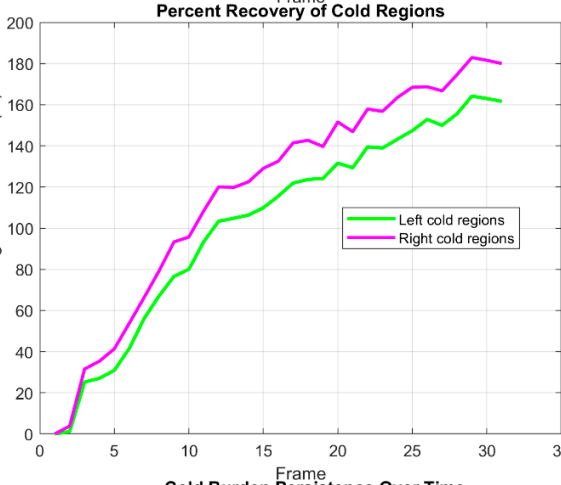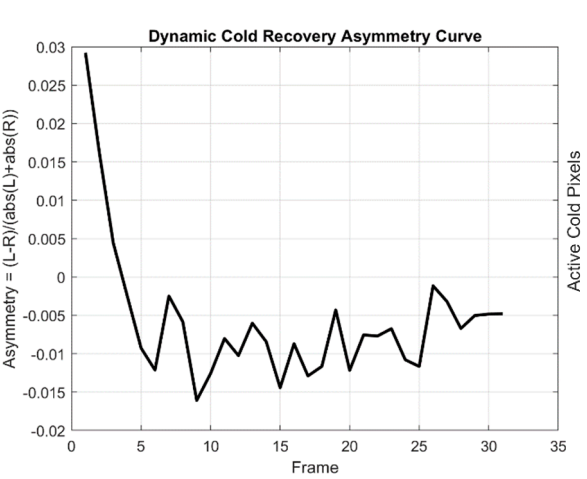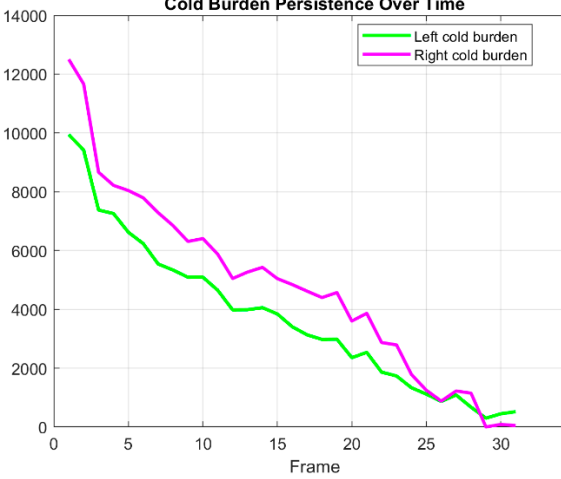

## H09

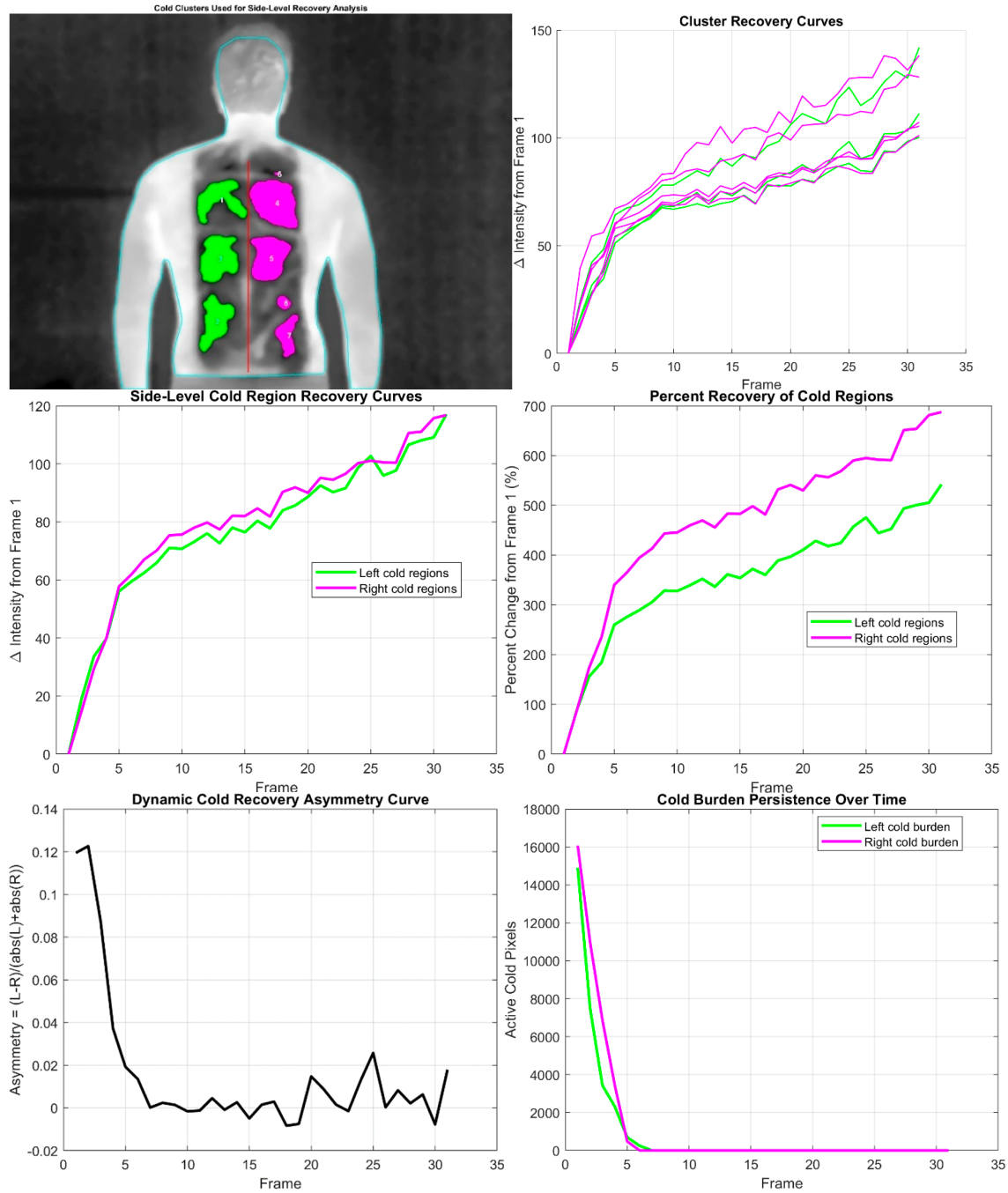

H10

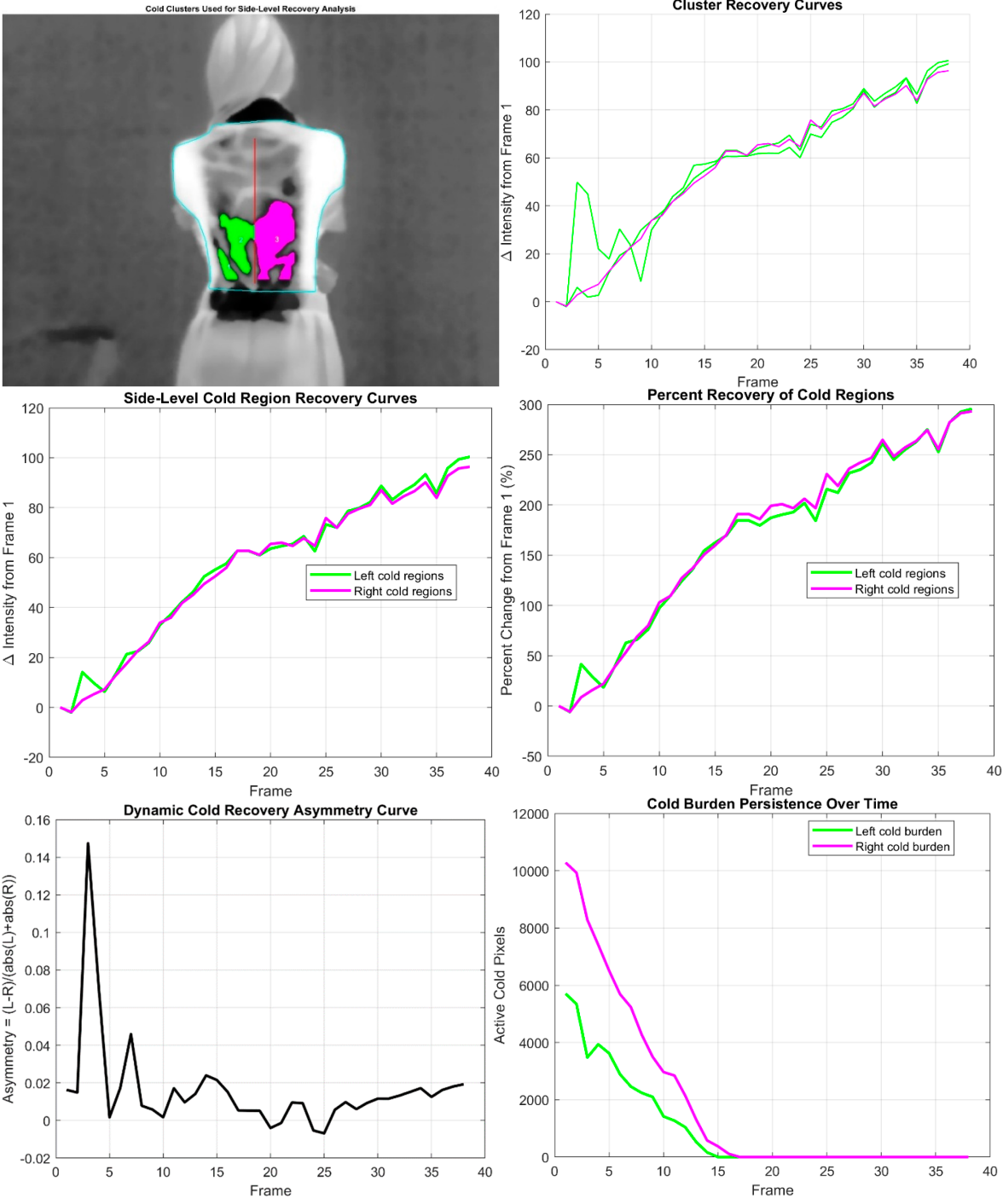

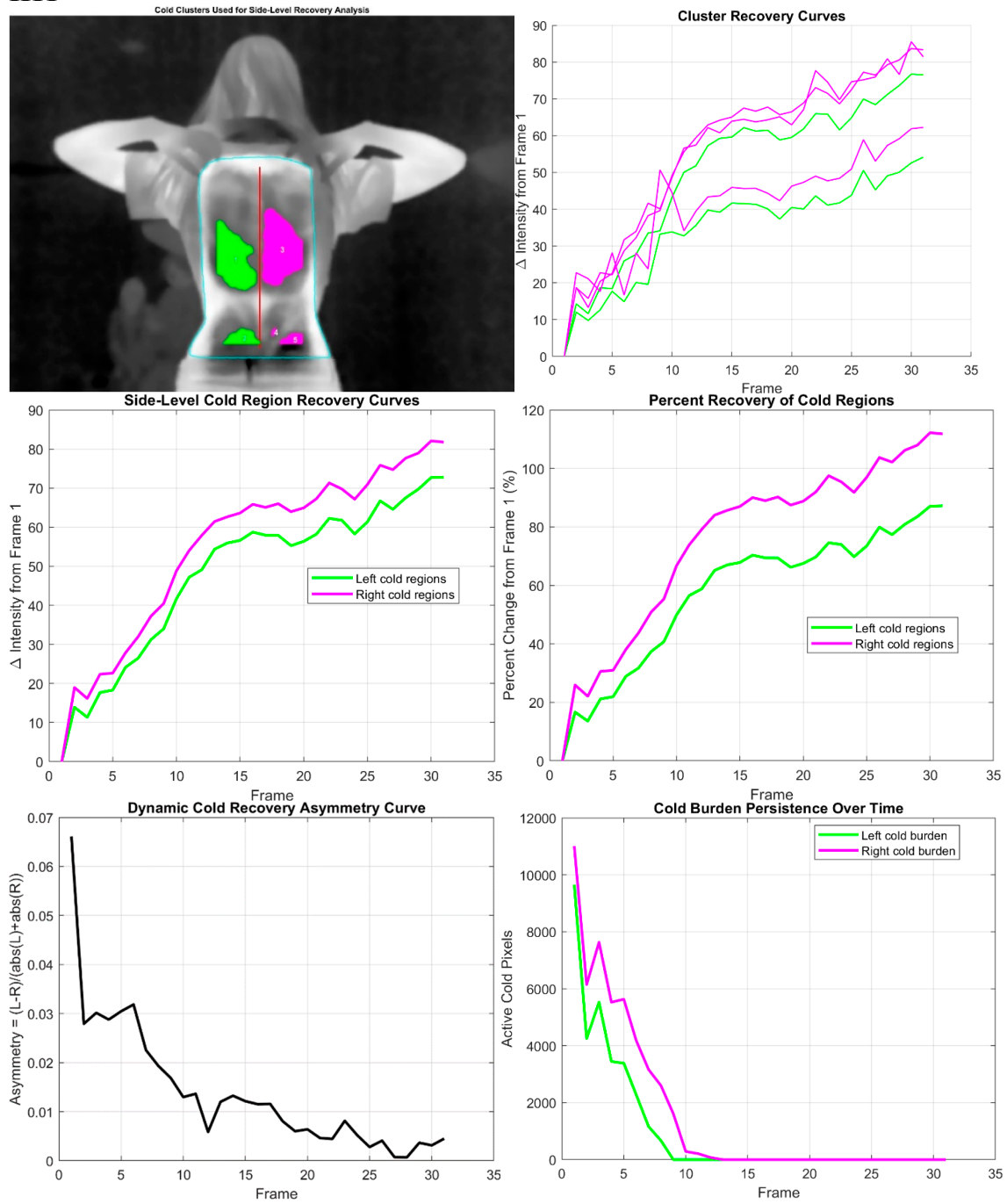

## H12

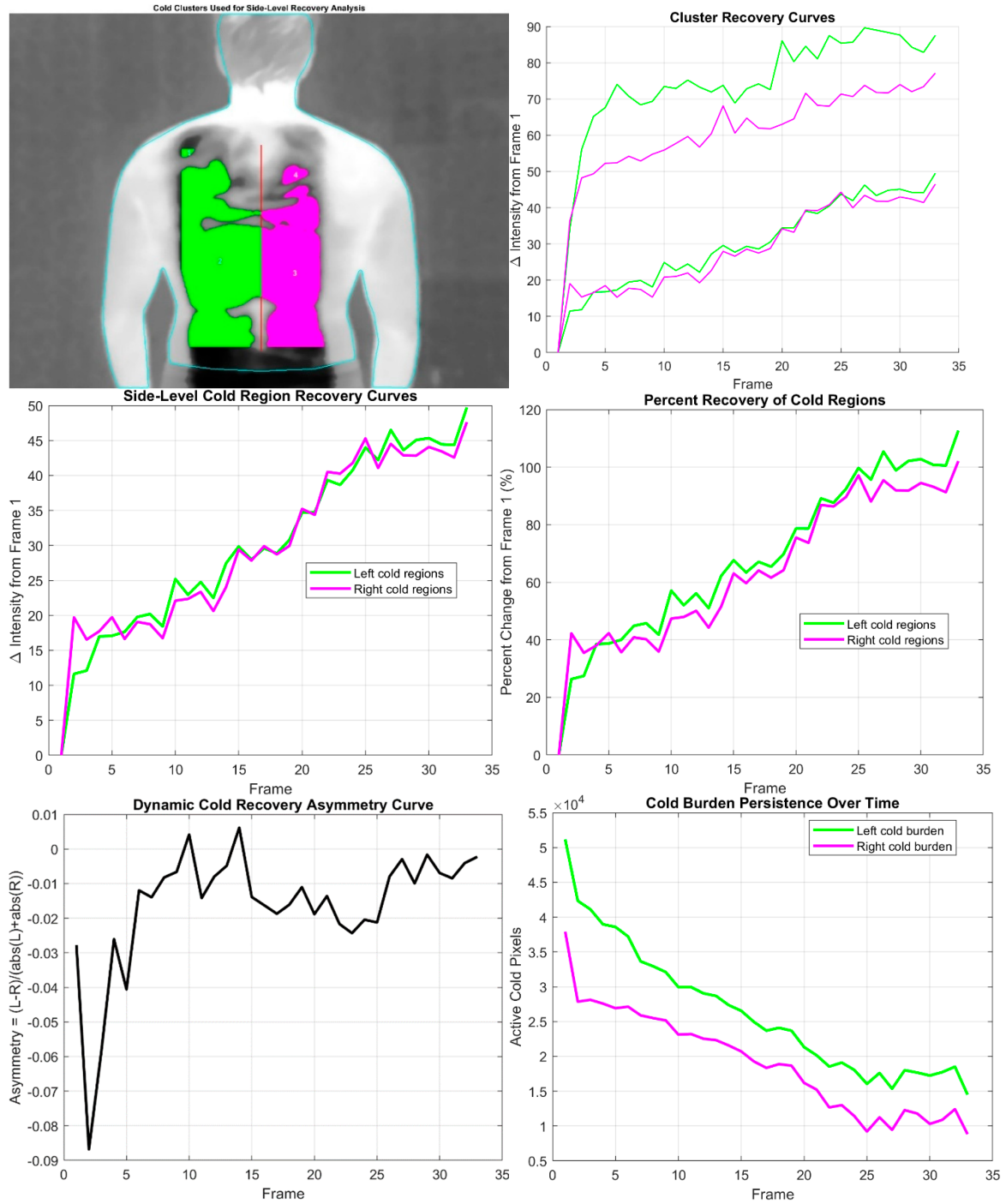

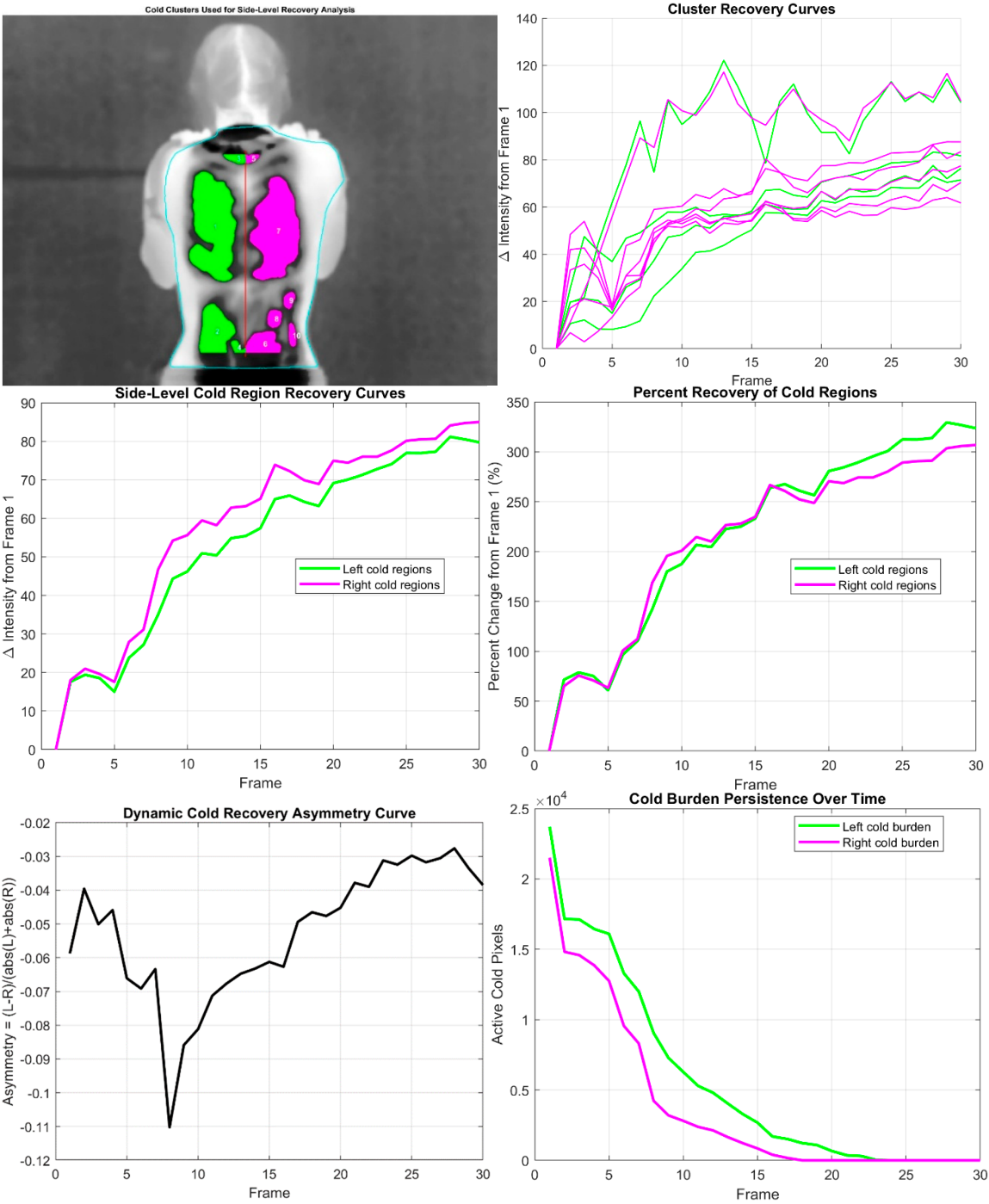

S01

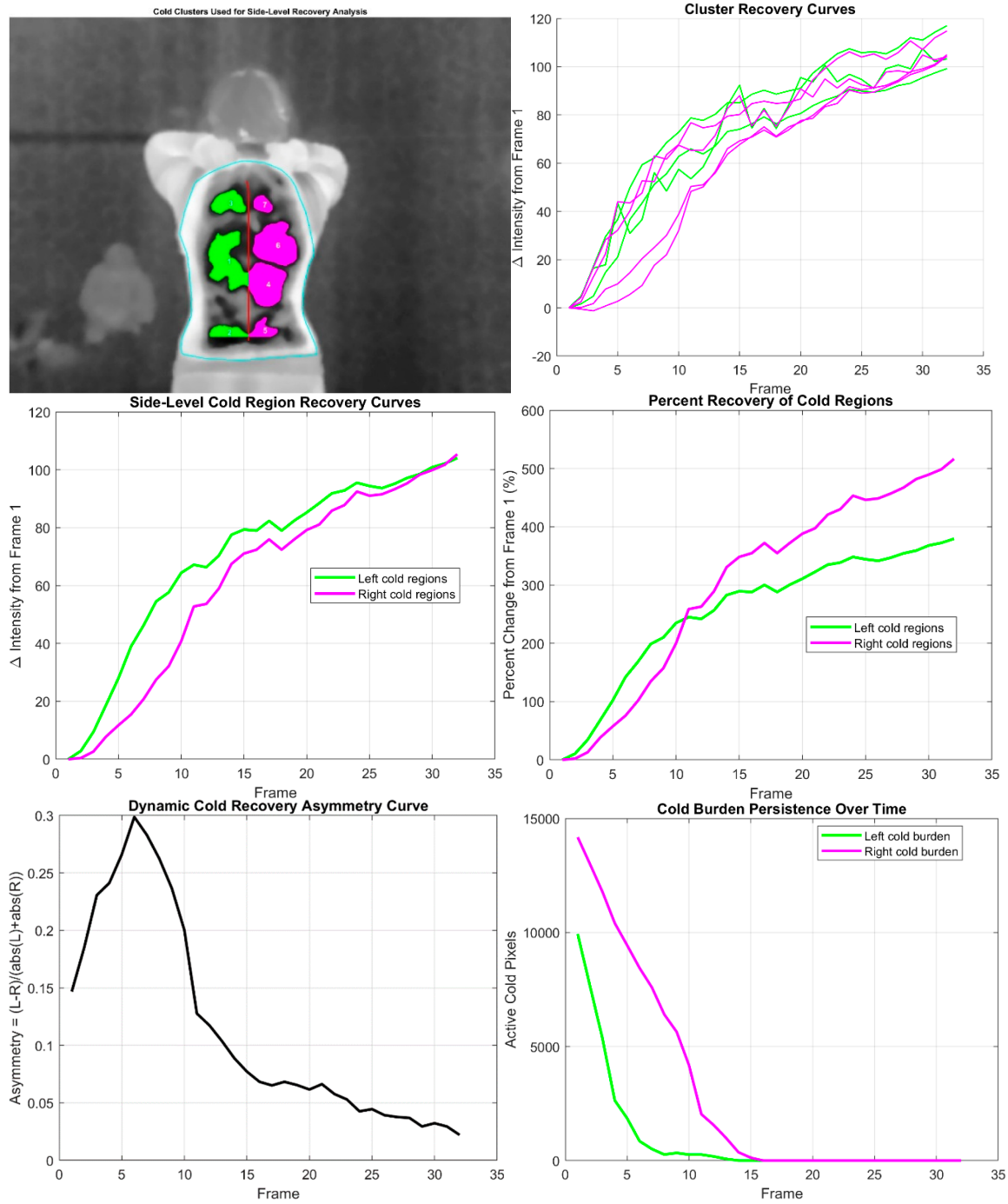

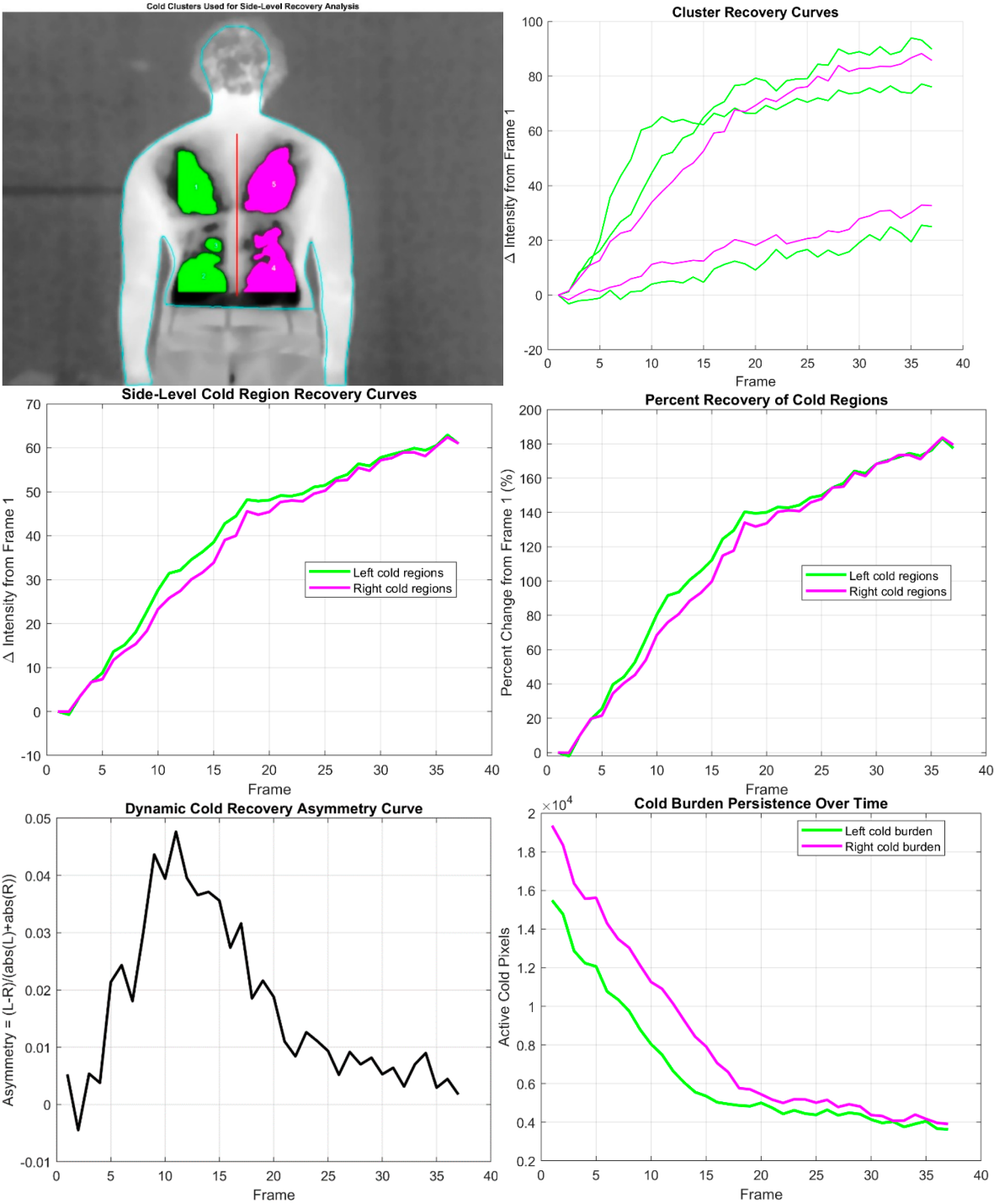

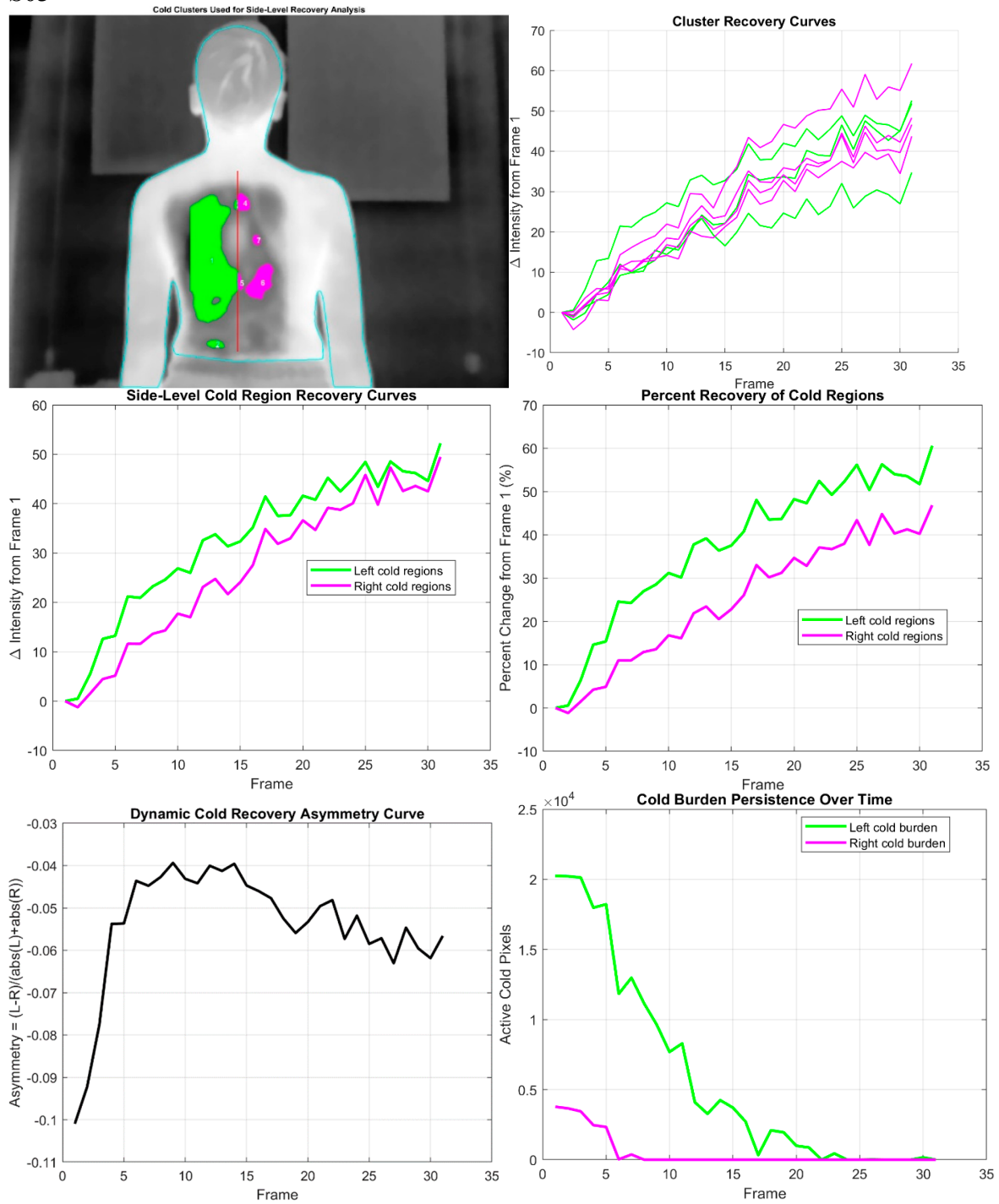

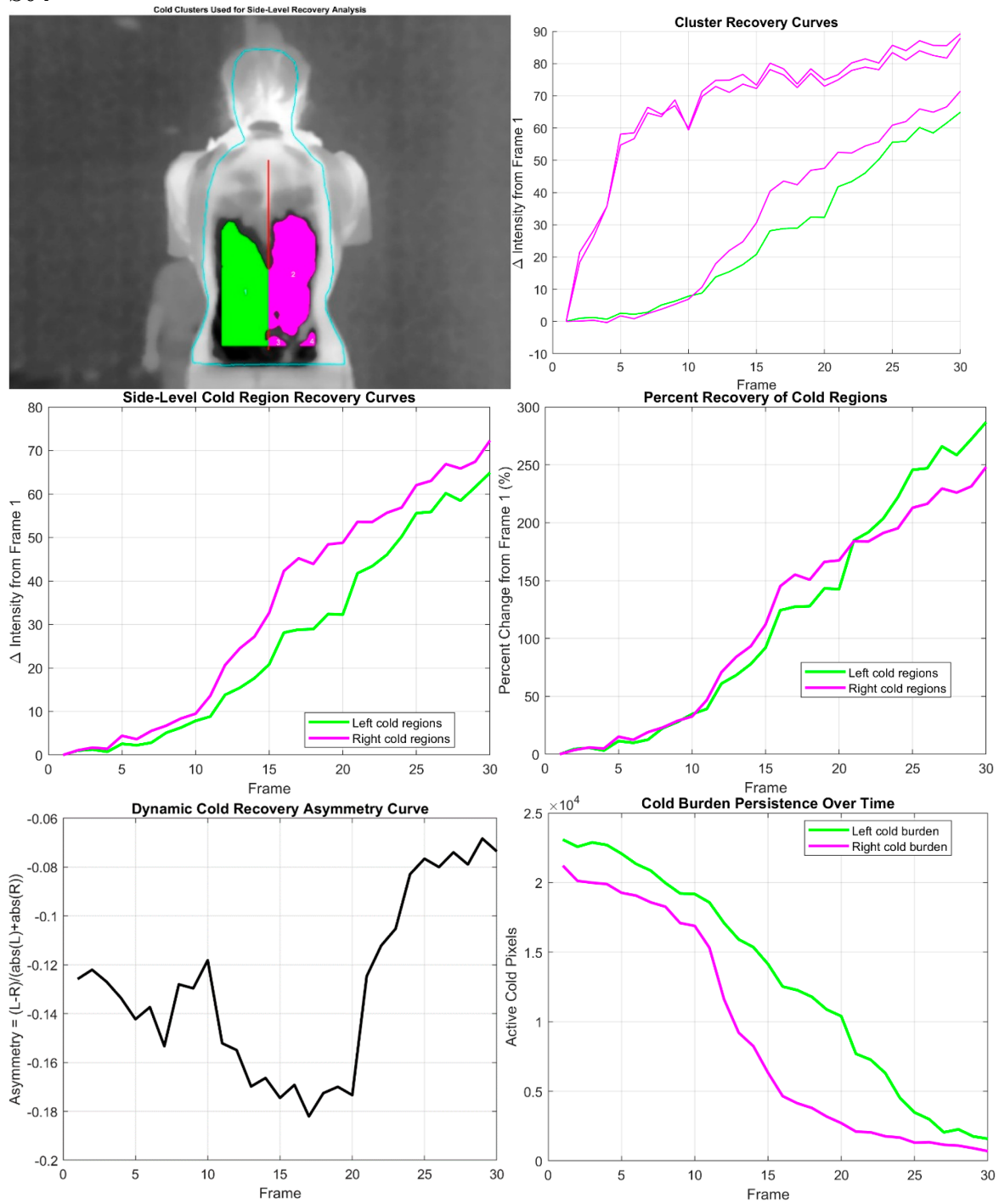

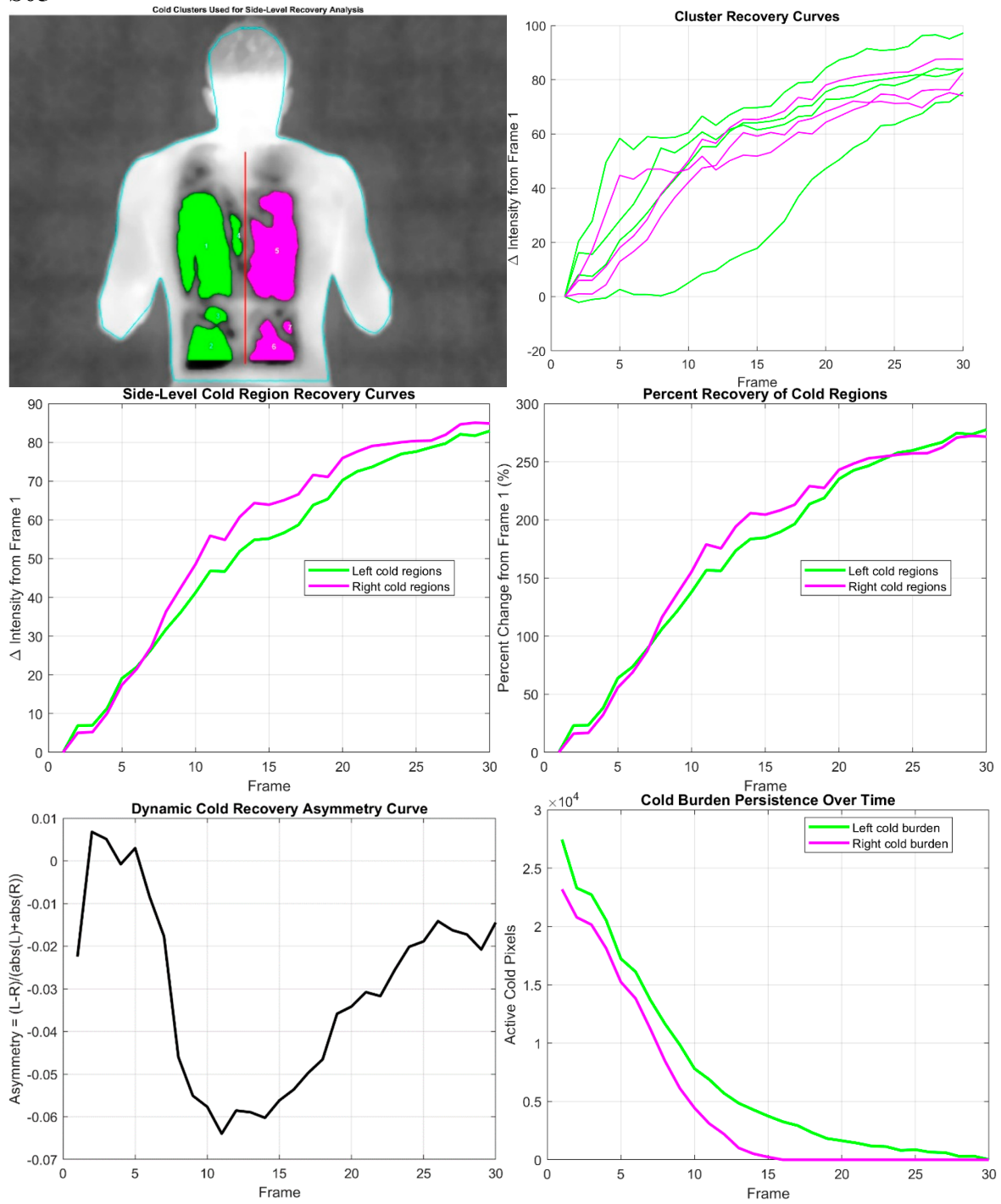

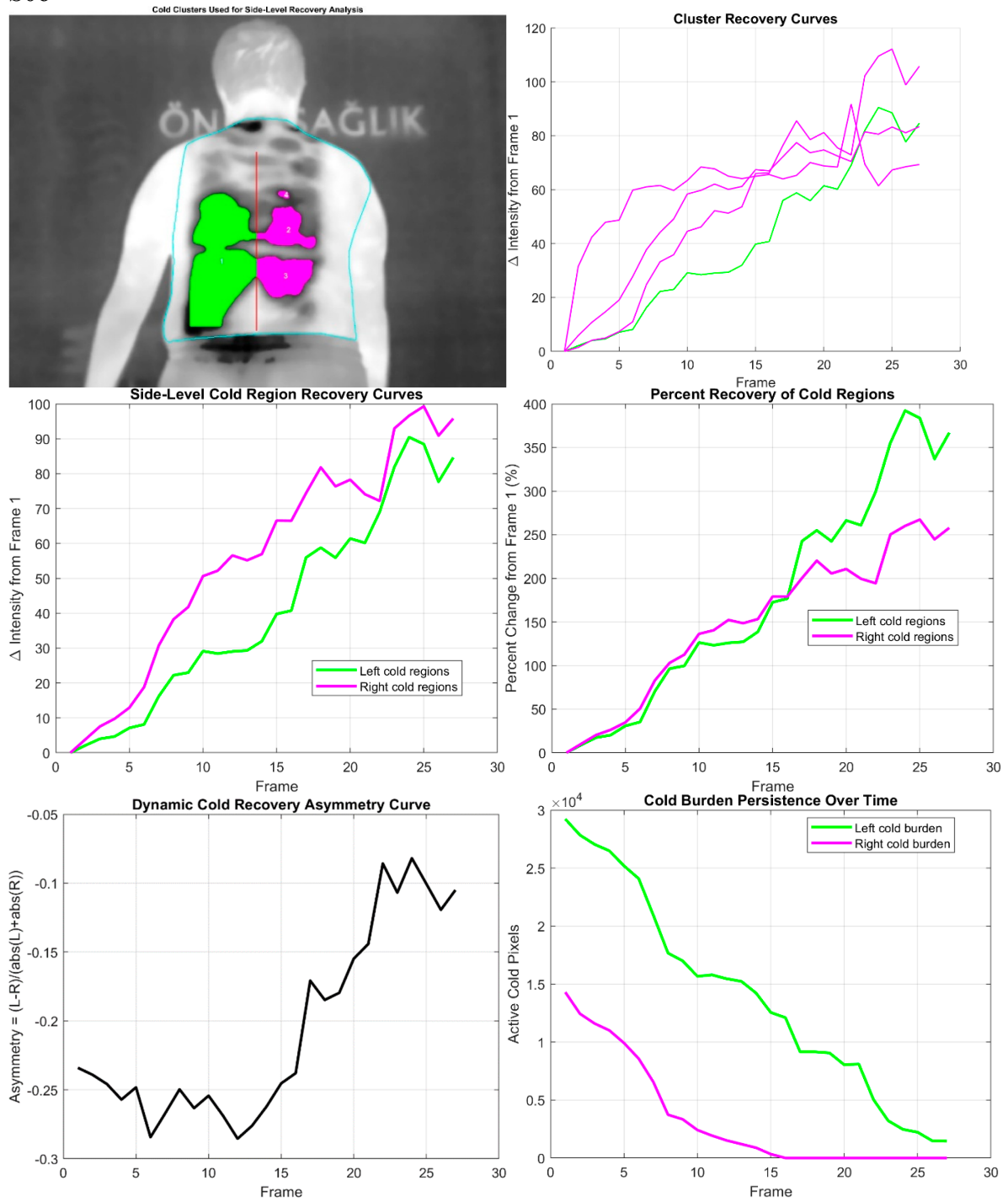

S07

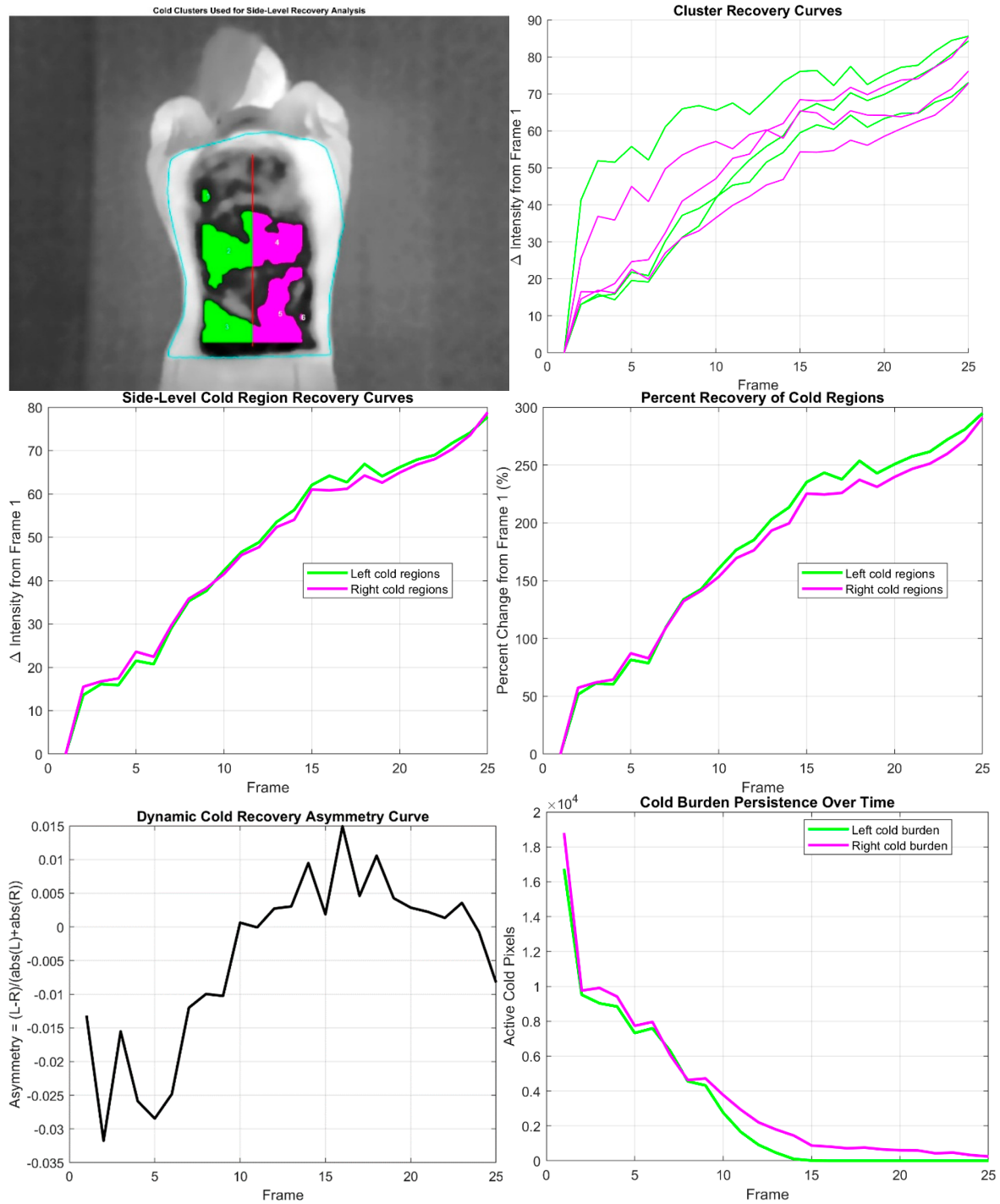

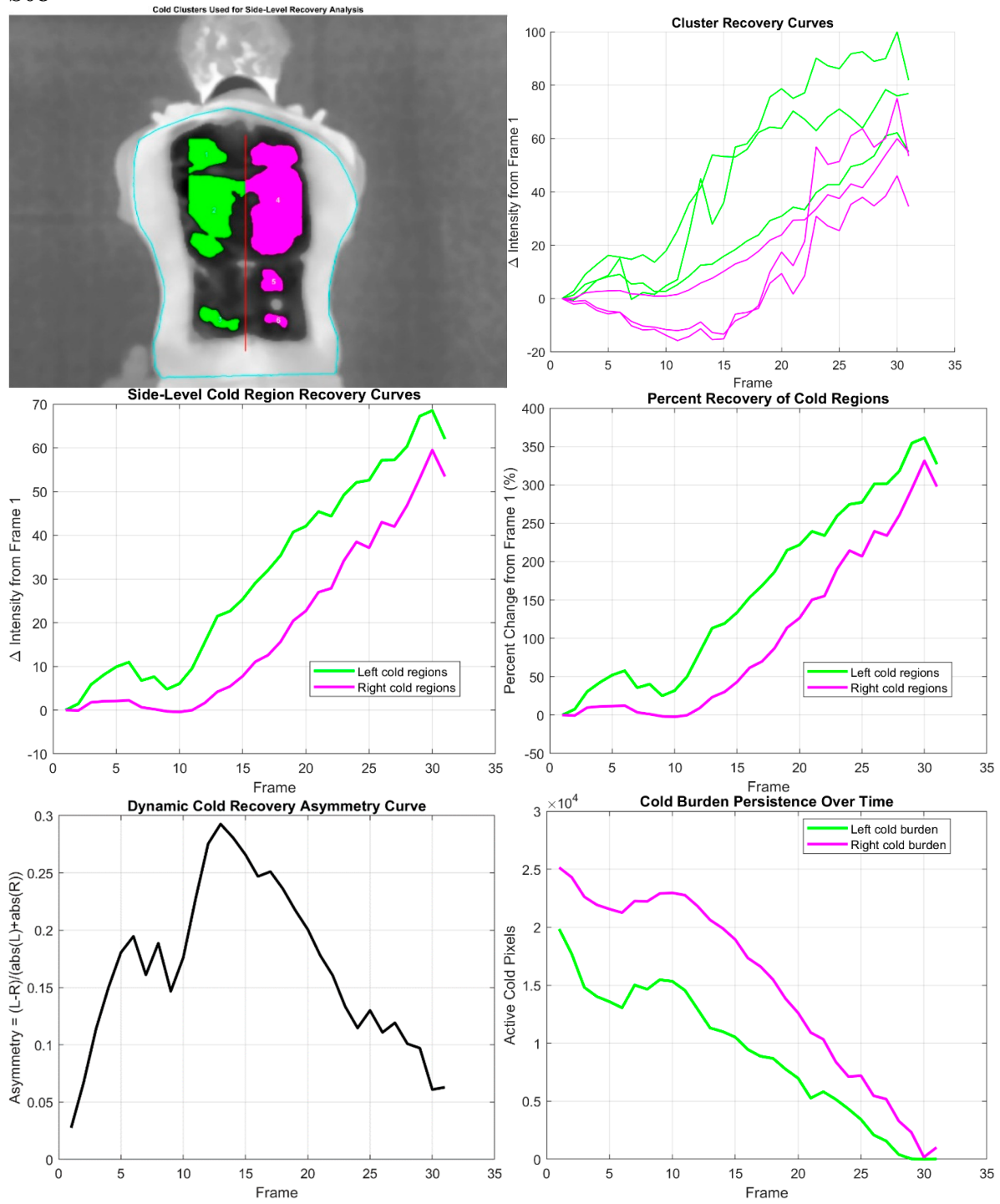

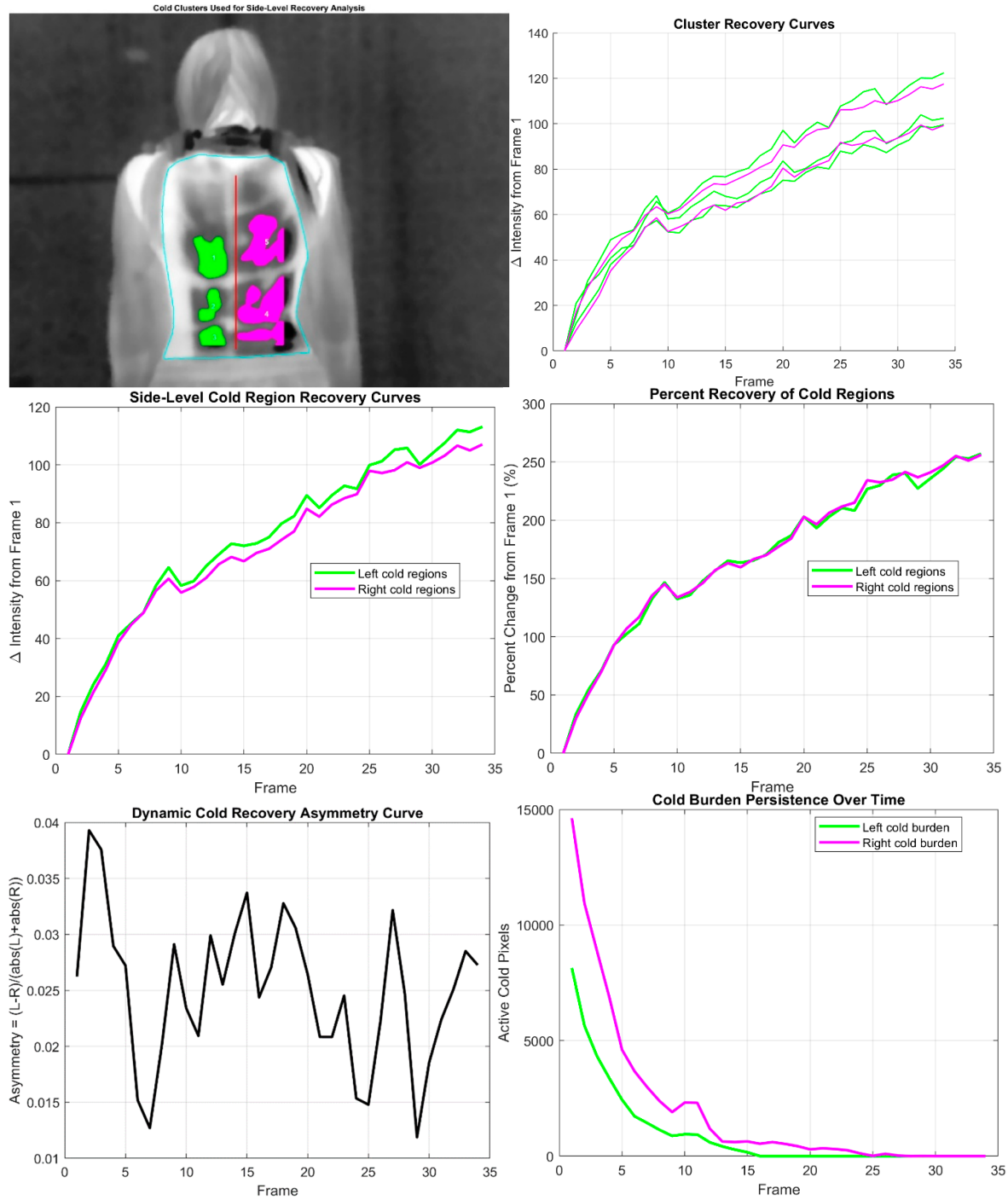

S10

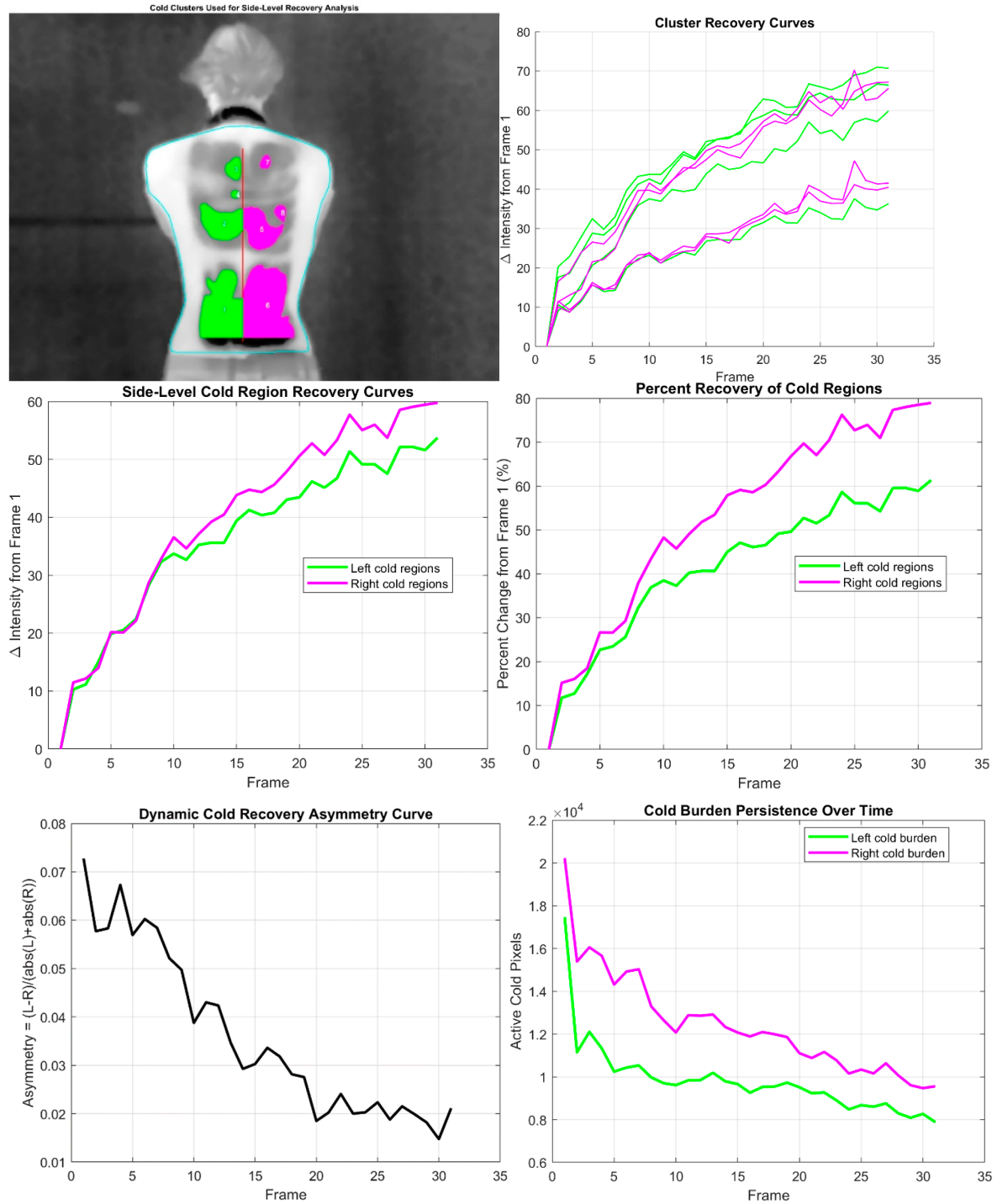

ct.

Supplement: Supplementary file 1 [file jcm-15-05720-s001.zip › jcm-4384695-supplementary.pdf]
